# Chemotherapy-induced alterations in miRNA expression and their prognostic implications in ovarian cancer

**DOI:** 10.3389/fonc.2025.1580565

**Published:** 2025-08-22

**Authors:** Samruddhi Ranmale, Pavan Kumar, Hemant Tongaonkar, Sanket Mehta, Vashishth Maniar, Jayanti Mania-Pramanik

**Affiliations:** ^1^ Department of Infectious Biology, ICMR-National Institute for Research in Reproductive and Child Health, Mumbai, India; ^2^ Urologic and Gynecologic Oncology Department, P. D. Hinduja Hospital and Medical Research Centre, Mumbai, Maharashtra, India; ^3^ Surgical Oncology Department, Saifee Hospital, Mumbai, Maharashtra, India

**Keywords:** miRNA, ovarian cancer, clinical specimens, chemotherapy, expression profiling, Biomarker, India

## Abstract

**Introduction:**

Ovarian cancer has a high mortality rate due to late diagnosis, relapse and chemoresistance. miRNAs play a major role in tumorigenesis as well as chemoresistance. Hence, we undertook a study, to evaluate the differential expression of miRNAs in clinical specimens of ovarian cancer patients that may highlight the effect of chemotherapy and their role in predicting survival outcomes.

**Methods:**

Clinical specimens were collected from n=127 participants comprising chemo-naive, chemo-treated ovarian cancer patients and healthy women as controls for the study. miRNA expression was evaluated by quantitative real-time PCR analysis.

**Results:**

Our results indicate an upregulation of miR-182 and miR-130a in the serum of treated ovarian cancer patients, which may be an effect of chemotherapy. Tissue levels of miR-182 and miR-106a were elevated in patients with advanced-stage disease. Elevated tissue expression of miR-106a was also associated with poor chemotherapy response and early relapse, while miR-200a was elevated in metastatic patients and linked to early relapse. However, reduced tumor suppressor miR-433 and miR-145 levels were observed in metastatic patients. Multivariate Cox regression identified serum miR-130a and tissue miR-20a as independent predictors of progression-free survival. A combined serum miRNA panel (miR-182, miR-106a, miR-23b) demonstrated diagnostic potential with an AUC of 0.743.

**Conclusion:**

The study highlights differential regulation of circulating and tissue miRNAs in Indian OC patients, emphasizing the selective retention of oncogenic miRNAs in tumors and release of tumor suppressive miRNAs into circulation. These findings support the utility of miRNAs as diagnostic and prognostic biomarkers in OC.

## 1 Introduction

Ovarian cancer (OC) ranks 8^th^ and accounts for 4.4% of all cancer-related deaths in females worldwide (1). Due to the asymptomatic nature of the disease, most OC cases are diagnosed at an advanced stage. Upon diagnosis, primary de-bulking surgery (PDS) followed by chemotherapy was regarded as the standard treatment for OC, as optimal de-bulking is directly associated with improved survival outcomes (2). Whereas in advanced OC with extensive tumor burden, neo-adjuvant chemotherapy succeeded by interval debulking surgery (IDS) has become the preferred treatment approach (3, 4). According to the CONCORD-3 data, the 5-year survival for ovarian cancer increased from roughly 28-36% in the early 2000s to approximately 30-47% by 2014 across 71 countries, which is far from optimal (5). Chemoresistance is considered a hurdle in the effective treatment of OC patients, contributing to higher relapse and mortality rates (6).

miRNAs are small non-coding RNAs involved in gene regulation either by translation repression/mRNA degradation (7). They control around 50% of all protein-coding genes and regulate several cellular processes (8). In cancer cells, the expression of miRNAs is altered, which can aid in tumor growth and metastasis (9). These changes could be attributed to regulation at the transcription level, post-transcriptionally, and by miRNA secretion (10). The latter is the most economical method as it does not involve the recruitment of various proteins, transcription factors, and other complex feedback mechanisms (11). miRNAs are versatile players as they can act either as oncomiRs or tumor suppressor miRNAs, depending upon their target mRNA (12). Emerging evidence suggests that tumors can selectively retain oncogenic miRNAs and export tumor suppressor miRNAs out of the cells to preserve particular stages of gene expression or growth (11, 13). Various mechanisms of miRNA selection have been reported, such as sequence-specific motifs, 3’-end modifications, etc. (13–15).

Moreover, the expression of miRNAs is dysregulated in response to chemotherapy and can predict prognosis (16). The role of miRNAs in the development of chemoresistance has been extensively studied in cell lines. However, only a few of these miRNAs have been validated in clinical OC specimens. miRNAs offer significant advantages over protein markers as they can be detected by PCR, enabling the development of multiplexed panels. Additionally, circulating miRNAs in the plasma and serum hold great potential as biomarkers for cancer (17, 18). In India, OC has emerged as one of the most common gynecological malignancies. However, there is a lack of reports on the association of miRNAs with treatment response monitoring in Indian women with OC.

Hence, we selected a panel of ten microRNAs (miR-145-5p, miR-182-5p, miR-200a-5p, miR-20a-5p, miR-23b-3p, miR-365a-3p, miR-433-3p, miR-106a-5p, miR-130a-5p, and miR-9-5p) based on a combination of differential expression analysis and supporting literature. Initial selection was guided by analysis of a publicly available dataset of serum miRNA expression from the GEO datasets (GSE79943). Differentially expressed miRNAs were then cross-referenced with published studies reporting their functional roles in ovarian cancer. Many of these miRNAs play significant roles in the development of chemoresistance of OC. Among these, miR-182, miR-130a, and miR-106a are oncogenic miRs reported to be involved in tumor proliferation, metastasis, and chemoresistance. Overexpression of miR-130a and miR-182 targets PTEN mRNA, potentially leading to cisplatin resistance in A2780 cells (19). Similarly, miR-106a expression can influence paclitaxel sensitivity in OC cells (20). Conversely, miR-145 and miR-433 are tumor suppressor miRNAs that regulate the proliferation of OC cells (21, 22). Altered expression of the tumor suppressor miRNAs miR-365, miR-23b, and miR-20a has been associated with poor survival outcomes (23, 24). Whereas oncogenic miR-200a and miR-9 are implicated in the invasion, metastasis, and chemoresistance in OC (25, 26).

Building on this background, we aimed to test the hypotheses that the levels of oncomiRs or tumor suppressor miRNAs in the circulation and tumors of OC patients are modulated by chemotherapy treatment. Additionally, we seek to determine whether these miRNA levels can serve as biomarkers for predicting treatment response and prognosis.

## 2 Materials and methods

### 2.1 Participant enrollment

Epithelial ovarian cancer (EOC) patients undergoing treatment at the P.D. Hinduja Hospital & Medical Research Centre, and Saifee Hospital, Mumbai, India during 2017–2021 were enrolled in the study. Before enrollment in the study, each participant provided written informed consent that had been authorized by the Institutional Ethics Committee (IEC) of ICMR-National Institute for Research in Reproductive and Child Health, as well as the ethics committees of both collaborative hospitals. A written informed consent form was also signed by all the healthy women enrolled in the study before blood collection. Blood and primary ovarian tumor specimens were collected from OC patients, while only blood was collected from healthy women who served as controls. All the tissue specimens were confirmed by immunohistochemistry to be primary ovarian tumors of high grade serous epithelial OC by a trained histopathologist. Blood specimens were collected from a few OC patients during their first line chemotherapeutic regimen (Carboplatin + paclitaxel) up to 3 cycles.

### 2.2 RNA extraction

The primary ovarian tumor tissues obtained during surgery were snap-frozen and preserved at -80°C. Total RNA extraction was done using the TRIzol (Invitrogen) extraction method according to the manufacturer’s protocol. For tissue samples, RNA quality was assessed using Nanodrop spectrophotometry. Samples were included only if they met minimum purity thresholds: A260/280 ratio ≥ ≥1.9-2.0 and A260/230 ratio ≥ 2.0. Blood specimens were collected in BD Vacutainer^®^ Serum tubes, incubated at ambient temperature for 30 minutes to facilitate coagulation and then centrifuged at 2000 rpm for 15 minutes. The separated serum was aliquoted and stored at -80°C until further use. Serum samples were assessed for hemolysis by measuring absorbance at 414 nm. Samples with optical density >0.2 and indicative of possible hemolysis were excluded from the study. RNA isolation from serum was done by using miRNeasy Serum/Plasma Advanced Kit (Qiagen) by following the manufacturer’s protocol. For serum samples, we used 400µl of serum to ensure good RNA concentration. After RNA extraction, quantification was performed using both Nanodrop spectrophotometry and Qubit fluorometry for accuracy. Only samples with A260/280 ratio ≥1.9-2.0 and A260/230 ratio ≥ 2.0 and detectable RNA levels (≥6.0 ng/µL) were included. The isolated RNA was reverse transcribed to cDNA using miScript II RT kit (Qiagen) according to the manufacturer’s protocol. For each cDNA synthesis reaction, a total of 50 ng RNA was used for serum samples, while 100 ng RNA was used for tissue samples. The cDNA was then used for quantitative real-time PCR experiments.

### 2.3 Real-time PCR

The expression of miRNAs was quantified by real-time PCR using Quantinova SYBR green PCR kit (Qiagen). Each miRNA was quantified in triplicate assays, and a real-time PCR machine (CFX96 touch, Bio-Rad) was used for detection. The relative expression levels of miRNA were analyzed by using the ΔCq method. Normalization was carried out with U6 snRNA (control). The primer sequences are given in **Table 1**.

**Table 1:** Details of primer sequences used for quantitative real-time PCR analysis.

MiRNA	Sequence
miR-182-5p forward	5’-GTTTGGCAATGGTAGAACTCA-3’
miR-433-3p forward	5’-CATGATGGGCTCCTCG-3’
miR-145-5p forward	5’-GTCCAGTTTTCCCAGGAATC-3’
miR-106a-5p forward	5’-GAAAAGTGCTTACAGTGCAG-3’
miR-9-5p forward	5’-GCAGTCTTTGGTTATCTAGCTG-3’
miR-200a-5p forward	5’-TAACACTGTCTGGTAACGATGT-3’
miR-23b-3p forward	5’-AGATCACATTGCCAGGGAT-3’
miR-365a-3p forward	5’-GCAGTAATGCCCCTAAAAATCC-3’
miR-130a-5p forward	5’-GGCTCTTTTCACATTGTGC-3’
miR-20a-5p forward	5’-CGCAGTAAAGTGCTTATAGTG-3’
Universal reverse	5’-GAATCGAGCACCAGTTACGC-3’
U6snRNA forward	5’- CTCGCTTCGGCAGCACA-3’
U6snRNA reverse	5’-AACGCTTCACGAATTTGCGT-3’

### 2.4 Statistical analysis

The expression level of miRNAs was compared between the different groups using the Man-Whitney U test at 95% confidence interval. Longitudinal analysis of follow-up samples was conducted on 16 patients with repeated measurements at four time points: a baseline sample collected at the time of primary cytoreductive surgery prior to any intervention, and three subsequent samples collected during consecutive adjuvant chemotherapy cycles. These data were analyzed using a mixed-effects model ANOVA with restricted maximum likelihood (REML) estimation. The data are represented as Mean ± SEM (Standard Error of the Mean), and the distribution of miRNA expression in the study population is provided in **Supplementary Table S1**. The correlation analysis of the clinicopathological features of OC patients with the expression of miRNAs in the serum and tissue specimens was done using Spearman’s rank correlation analysis at a 95% confidence interval. Kaplan-Meier method was used for survival analysis. Survival curves were plotted and compared by log-rank test for statistical analysis. Receiver operating characteristic (ROC) curve analysis was performed to evaluate the diagnostic utility of individual miRNAs. The area under the ROC curve (AUC), sensitivity, and specificity were calculated for each miRNA. A composite ROC curve was also generated by combining the top-performing miRNAs using binary logistic regression to assess whether diagnostic accuracy could be improved. The prognostic relevance of miRNA expression and clinical features with progression-free survival (PFS) was assessed by a multivariate Cox proportional hazards regression analysis performed using R (version 4.3.2) and the survival package. The analysis was conducted separately for tissue and serum miRNA expression profiles. Covariates included the expression levels of all 10 selected miRNAs, age, treatment mode, FIGO stage, CA-125 levels, lymph node metastasis, presence of metastatic cells in ascites, and Chemo response score (CRS). Hazard ratios (HRs), 95% confidence intervals, and p-values were calculated for each covariate, and model performance was evaluated using concordance statistics and likelihood ratio testing. For statistical analysis, GraphPad Prism software (Version 9.5.1) was used. The threshold for significance was set at p-value <0.05.

## 3 Results

In this study, we enrolled 127 participants, of whom 83 were with serous epithelial OC patients and 44 healthy women as controls. The median age of the enrolled OC patients was 54 years (Range 32–81 years), with 18 premenopausal and 65 postmenopausal women, while the median age of healthy controls was 51 years (Range 30–67 years), including 8 premenopausal and 36 postmenopausal women. There was no significant difference in age between patients and healthy controls (p = 0.9065). The healthy controls were arbitrarily selected women with no known malignancies or major co-morbidities.

Blood specimens (n = 83) were collected from ovarian cancer (OC) patients at the time of enrollment. Among them, 25 patients were in the chemo-naive group, meaning they were newly diagnosed and scheduled directly for primary cytoreductive surgery without receiving any prior treatment; both blood and tissue samples were collected intraoperatively. Fifty-eight patients were classified into the chemo-treated group; they received three cycles of neoadjuvant chemotherapy (paclitaxel and carboplatin) prior to surgery, and their blood and tissue samples were also collected at the time of surgery. Additionally, a follow-up group consisted of 16 OC patients from whom serial blood samples were collected at four time points: initially at the chemo-naive stage and then during each of the three subsequent scheduled chemotherapy cycles (n = 16 × 4 total serum samples). Blood samples were also collected from healthy women (n = 44) enrolled as controls at a single time point for comparison.

Tumor tissues (n = 76) were collected from OC patients at the time of surgery. All the tissue specimens were confirmed by immunohistochemistry to be serous EOC by a trained pathologist. Some of the collected tissues (n = 20) were excluded from the study due to inadequacy or poor quality of extracted RNA. Specifically, 2 samples had insufficient tissue quantity, 5 samples exhibited low RNA yield, 7 samples showed poor RNA purity based on A260/230 ratios, and 6 samples contained necrotic tissue or high fat content.

We enrolled 83 women diagnosed with high-grade serous epithelial ovarian cancer. Around 13% (n = 11) of patients were diagnosed in the early stage (FIGO I), whereas more than 69% (n = 58) of patients were diagnosed at an advanced stage. These results highlight that the rate of early diagnosis of OC is very poor in the urban Indian population.

The presence of tumor cells in the lymph nodes is an indication of metastasis and is often linked with poor survival of OC patients. In our study, 46% (n = 39) of the total patients had lymph node metastasis these patients were more likely to be in FIGO stages III and IV (83%, n = 32) than in stages I and II (17%, n = 7). Similarly, among the 27 patients that had the presence of tumor cells in their ascites, only n = 5 patients belonged to stages I & II. The presence of metastasis in the lymph nodes or the peritoneal cavity further affects the prognosis of the disease. These findings indicate the need for developing early diagnostic and prognostic strategies for improving the survival outcome of OC patients. The detailed patient characteristics are given in **Table 2**.

**Table 2:** Clinicopathological characteristics of ovarian cancer patients enrolled in the study.

Parameters	Criteria	n	%
Primary Ovarian cancer patients		83	100
Age		54 years (Range 32–81 years)	
FIGO stage	I	11	13.25
	II	25	30.12
	III	44	53.01
	IV	3	3.61
Grade	High	83	100
Lymph node metastasis	Present	39	46.98
	Absent	44	53.01
Fluid cytology	Present	27	32.53
	Absent	40	48.19
	Unknown	16	19.27
Chemotherapy (Carboplatin + Paclitaxel)	Chemonaive	25	30.12
	Treated with 3 cycles of first line chemotherapy	58	69.87
Chemo response score	CRS 1	18	21.68
	CRS 2	25	30.12
	Unknown	15	18.07
Median CA-125 (U/ml)	Before treatment	736.8 U/ml	
	After treatment	25.5 U/ml	
Recurrence	Yes	42	50.6
	No	41	49.39
	Death due to disease	9	10.84
Healthy women	Controls	44	100
Age		51 years (Range 30–67 years	

### 3.1 Differential effect of chemotherapy on the expression of miRNAs in OC patients

**Figure 1:**
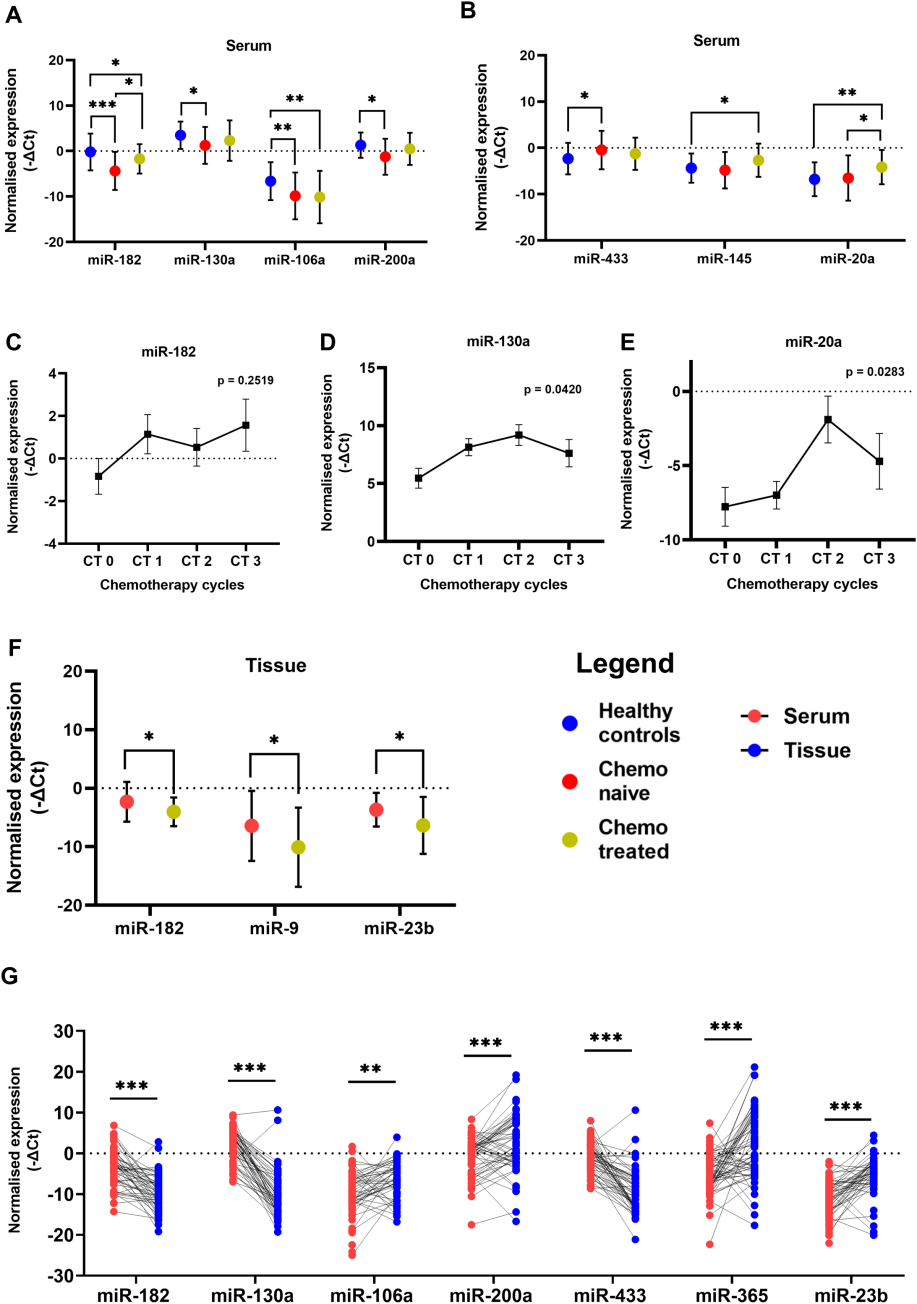
Relative levels of miRNAs in serum of patients with ovarian cancer and healthy women **(A)** Oncogenic miRNAs miR-182, miR-130a, miR-106a and miR-200a **(B)** Tumor suppressor miR-433, miR-145 and miR-20a. Relative levels of **(C)** miR-182, **(D)** miR-130a, and **(E)** miR-20a in paired serum samples collected from chemo-naive and post-chemotherapy cycle follow-up time points in ovarian cancer patients (n = 16). **(F)** The relative miRNA levels in tumor tissues of chemo naive and chemotherapy-treated ovarian cancer patients. (Data are presented as Mean ± SEM) **(G)** Wilcoxon paired analysis between serum and tissue specimens from each individual ovarian cancer patient. *p < 0.05, **≤0.01, ***≤0.001.

In this study, we evaluated the expression of the selected miRNAs in the serum of chemo naïve OC patients and treated patients along with a control group of healthy women. Among these selected miRNAs; miR-182, miR-9, miR-130a, miR-200a and miR-106a act as oncomiRs, while miR-433, miR-145, miR-20a, miR-23b and miR-365 are considered as tumor suppressor miRNAs. The serum expression of miR-182 was downregulated in both groups of OC patients, chemo-naive (p = 0.0005) and chemo-treated (p = 0.0378), compared with healthy controls. However, the expression of miR-182 was significantly high (p = 0.0460) in the serum of patients treated with chemotherapy compared to chemo-naive OC patients (**Figure 1A**).

To further investigate these observations, we collected follow-up serum specimens from patients at intervals of every chemotherapy cycle up to 3^rd^ cycle. The expression of miR-182 was not altered significantly at different intervals although a trend of upregulation is evident in the serum collected during their follow-up (**Figure 1C**). In tumor tissues there was a significant decrease in miR-182 expression (p = 0.0304) of patients treated with three cycles of chemotherapy compared to chemo naive patients (**Figure 1F**). There are several reports of miR-182 which acts as an oncomiR in different cancers including OC. It is possible that miR-182 is released by normal cells in healthy women, and this release is suppressed by tumorigenesis. But after chemotherapy and reduction in tumor burden, the levels of miR-182 are again elevated in the serum. Hence, these results may be suggestive of a release of oncogenic miRNA-182 by the tumor cells upon treatment with chemotherapy.

Similarly, the expression of oncogenic miR-130a (p = 0.0171) and miR-200a (p = 0.0381) (**Figure 1A**) was significantly lower in the serum of chemo naive OC patients compared to healthy women. In the follow-up group of patients, the expression of miR-130a (**Figure 1D**) was significantly increased upon treatment with chemotherapy. The expression of oncogenic miR-106a was lower in the serum of both groups of OC patients compared to healthy women (p = 0.0098, p = 0.0019), suggesting chemotherapy does not affect its expression/release (**Figure 1A**), while there is a significant impact on the expression of miR-182 and miR-130a.

In the case of miR-433, a higher level of serum expression was evident in chemo-naive patients compared to healthy controls (p = 0.0372) (**Figure 1B**). However, its expression was not significantly altered in the serum (**Figure 1B**) as well as tissue (**Supplementary Figure S2B**) of the treated group of patients. The expression of miR-145 (p = 0.0406) and miR-20a (p = 0.0025) (**Figure 1B**) was higher in the serum of chemo-treated patients compared to healthy women. miR-20a expression is also higher (p = 0.0470) in chemo-treated compared to chemo naïve patients (**Figure 1B**). Also, in the follow-up group of patients, the expression of miR-20a is elevated post-chemotherapy (**Figure 1E**). In the tumor tissues of chemo-treated OC patients, the expression of miR-9 (p = 0.0443) and miR-23b (0.0433) was significantly reduced compared to that of chemo naïve patients (**Figure 1F**). These results suggest further elevation of miRNAs specifically miR-145 and miR-20a expression in serum, while expression of miR-9 and miR-23b was significantly reduced in tissues after chemotherapy.

### 3.2 Differential expression of miRNAs in paired serum and tissue specimens of OC patients.

**Figure 2:**
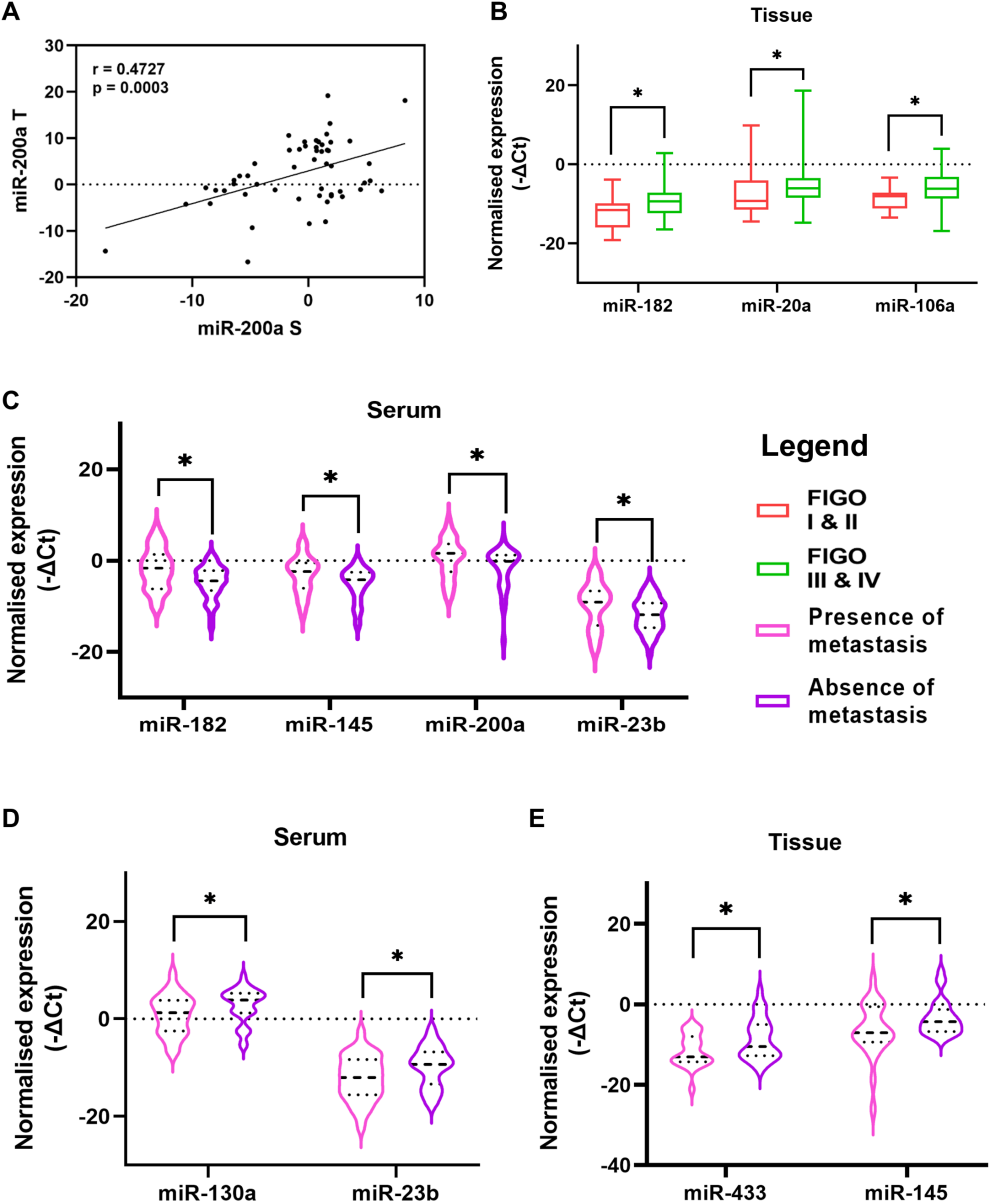
**(A)** Correlation of miR-200a expression in paired serum and tissue specimens of ovarian cancer patients **(B)** The difference between miRNA levels in tumor tissue of patients based on FIGO stage **(C)** Comparison of relative serum miRNA levels of patients with the presence or absence of metastatic cells in their ascitic fluid. Comparison between patients with presence or absence of lymph node metastasis **(D)** relative serum miRNA levels and **(E)** relative tissue miRNA levels (Data are presented as Mean ± SEM). *p < 0.05.

The expression levels of these miRNAs were explored by Wilcoxon paired non-parametric analysis in the paired serum and tissue specimens obtained from each patient. We found elevated levels of miR-182, miR-130a, and miR-433 in the serum of OC patients compared to the tissue levels. Whereas the expression of miR-106a, miR-200a, miR-23b, and miR-365 was higher in the tissue specimens compared to serum levels (**Figure 1G**). Also, the serum expression of oncogenic miR-200a was positively correlated with its expression in the tissue specimens of OC (**Figure 2A**).

### 3.3 Association of miRNA levels with clinicopathological characteristics

#### 3.3.1 Severity of the disease

The severity and progression of the disease are reported with the stage of the disease, presence of lymph node metastasis, dissemination of tumor cells or spheroids (clusters of tumor cells) in the peritoneal cavity, etc. We divided the patients based on these clinicopathological features and evaluated the expression level of the miRNAs under investigation. We observed that the expression of miR-182, miR-106a, and miR-20a was significantly upregulated in the tissues of OC patients diagnosed at an advanced stage compared to that of patients with FIGO stage I or II of the disease (**Figure 2B**).

A unique feature of OC is the accumulation of ascites in the peritoneal cavity. This ascites can carry tumor cells or spheroids that are detached from the primary tumor and can contribute to the peritoneal spread of the disease. The serum expression of four miRNAs, miR-182, miR-145, miR-200a, and miR-23b, was upregulated in patients who had the presence of metastatic cells in their ascites (**Figure 2C**). Similarly, the presence or absence of lymph node metastasis is a crucial factor that can affect the prognosis. In our study, the serum expression of miR-130a and miR-23b were lower in patients that had a presence of lymph node metastasis (**Figure 2D**). Similarly, two miRNAs, miR-433 and miR-145 were significantly reduced in the tissue of patients with lymph node metastasis (**Figure 2E**). These results suggest elevated expression of oncomiRs (miR-182, miR-106a) while a decrease in the expression of tumor suppressor miRs (miR-433 and miR-145) in tissues of patients at advanced stage or with lymph node metastasis.

#### 3.3.2 Chemo response score

**Figure 3:**
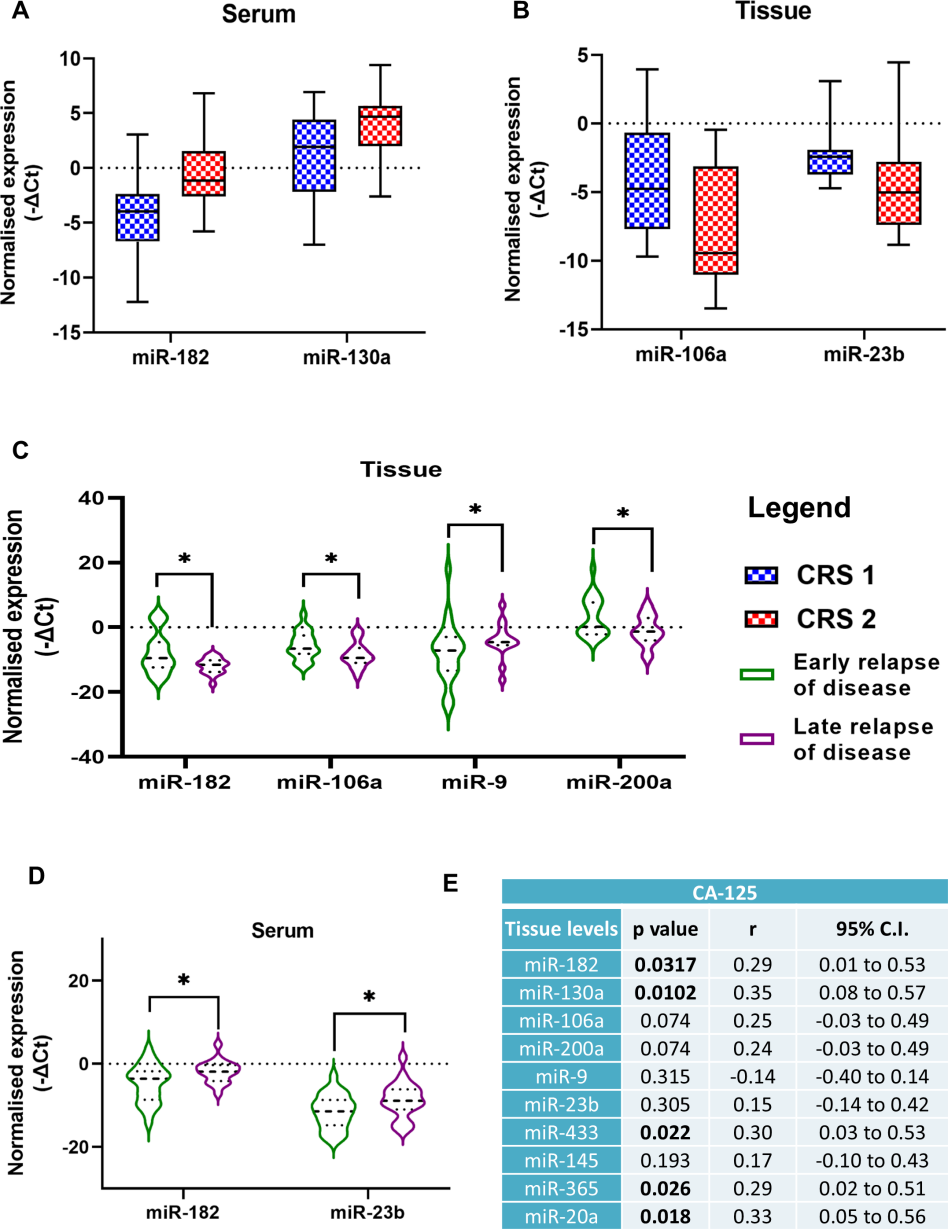
Comparison of miRNA expression levels in patients divided into two groups based on chemo response score **(A)** serum levels, **(B)** tissue levels. Comparison of miRNA expression levels in two groups of ovarian cancer patients in the **(C)** serum, and **(D)** tissue specimens based on early or late relapse of the disease (Data are presented as Mean ± SEM). **(E)** Correlation analysis of miRNA expression levels with serum CA-125 levels of ovarian cancer patients. *p < 0.05.

The prognosis of OC is dependent on factors like the spread and resectability of the disease, residual tumor burden post-surgery, and response to the chemotherapeutic drugs. One of these factors, i.e. the response of tumor cells to the chemotherapeutic regimen, can be estimated by the Histopathologist by estimating the extent of necrotic tumor tissue and is scored as CRS 1 (minimal or no response to chemotherapy) and CRS 2 (Good response to chemotherapy). The serum expression of miR-182 and miR-130a was lower in patients who had a poor response to chemotherapy (**Figure 3A**). Whereas, the tissue expression of miR-106a and miR-23b was lower in patients with a good response to chemotherapy (**Figure 3B**).

#### 3.3.3 Relapse of the disease

Among the women included in our study, 42 patients suffered from a relapse of the disease. Based on the median months of progression-free intervals (20 months), we divided patients into two groups, early and late relapse. OC patients with significantly lower expression of three miRNAs, miR-182, miR-106a, and miR-200a in their tumor tissues had a late relapse compared to patients with higher expression. Inversely, patients with higher miR-9 levels in their tissues had a late relapse of the disease (**Figure 3C**). The expression of miR-182 and miR-23b was elevated in the serum of patients with a late relapse of the disease (**Figure 3D**).

### 3.4 Association of clinicopathological characteristics with the survival of OC patients

**Figure 4:**
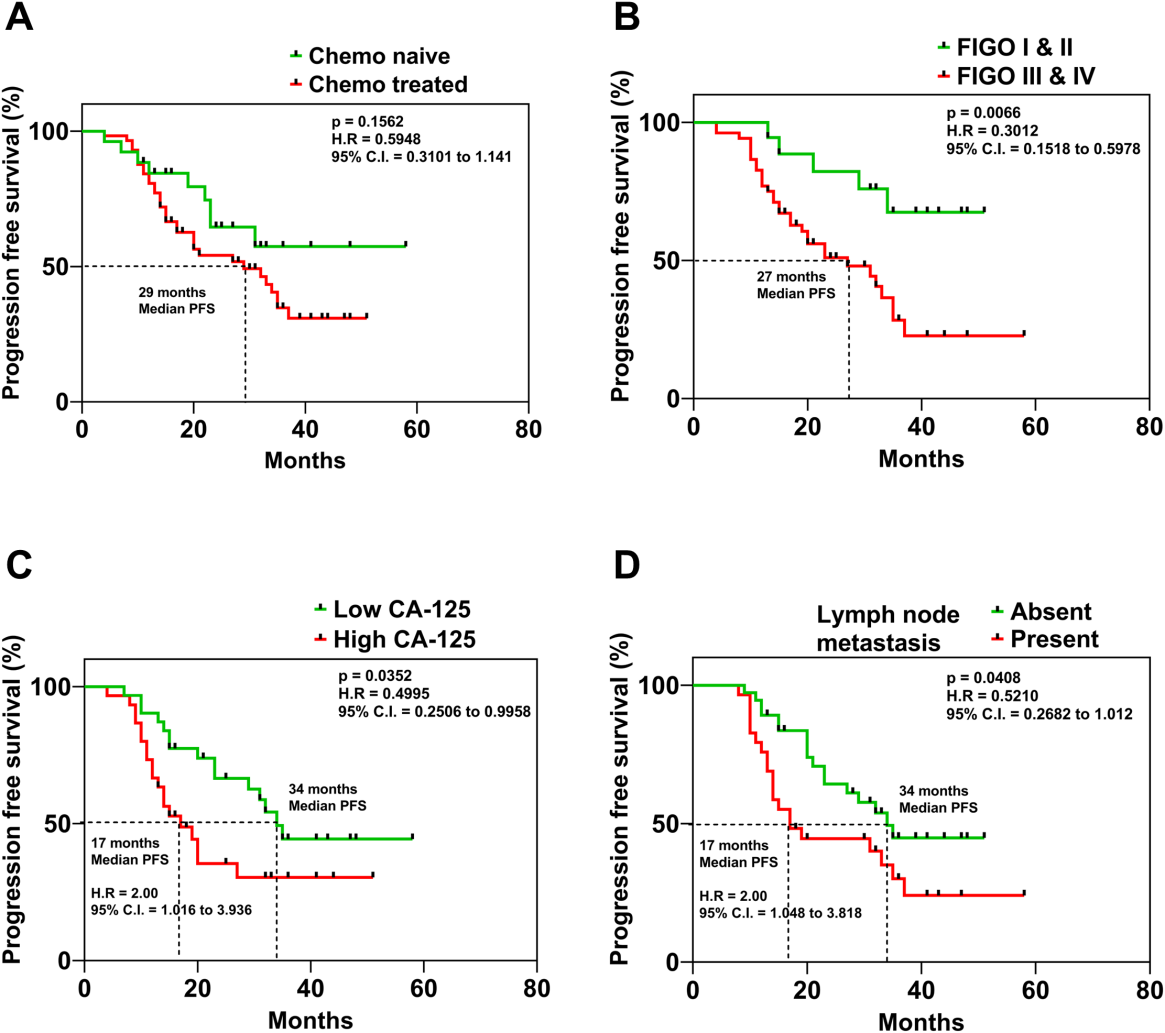
Kaplan Meier curves displaying the association of **(A)** mode of treatment, **(B)** FIGO Stage of the disease, **(C)** serum levels of CA-125 and **(D)** presence or absence of lymph node metastasis in ovarian cancer patients and their respective progression-free survival.

The correlation analysis of the clinical parameters of OC patients with the miRNA expression levels revealed that tissue levels of miR-182, miR-130a, miR-433, miR-365 and miR-20a were positively correlated with the CA-125 levels of the patients (**Figure 3E**). Patients with lower CA-125 levels had better progression-free survival (PFS) than those with higher CA-125 levels (p = 0.0325) (**Figure 4C**).

The Kaplan-Meier analysis was utilized to evaluate the association of prognosis with clinicopathological parameters. OC patients with a follow-up of <6 months were excluded from the analysis. The median overall survival (OS) time was 33 months (range 8–58 months), while the median PFS time was 20 months (range 7–58 months). Upon analysis, we found that the PFS of OC patients was not significantly different based on the treatment mode (**Figure 4A**). However, the timing of diagnosis was found to be a crucial factor influencing PFS, according to the study’s findings. The PFS of OC patients with (FIGO) stage I & II was significantly better compared to patients diagnosed with (FIGO) stage III & IV of the disease (p = 0.0066) (**Figure 4B**). These results emphasize the need for early detection of OC for ensuring better survival outcomes. On the other hand, patients with lymph node metastasis had a significantly lower PFS (p = 0.0408) compared to patients whose lymph nodes were negative for tumor cells (**Figure 4D**). Kaplan–Meier survival analyses were also performed to investigate the association between miRNA expression levels and survival outcomes. Patients were stratified into high and low expression groups for each miRNA in the panel. However, none of the miRNAs showed statistically significant correlations with progression-free survival and overall survival (**Supplementary Tables S2, S3**).

### 3.5 Diagnostic performance of individual and combined miRNAs

**Figure 5:**
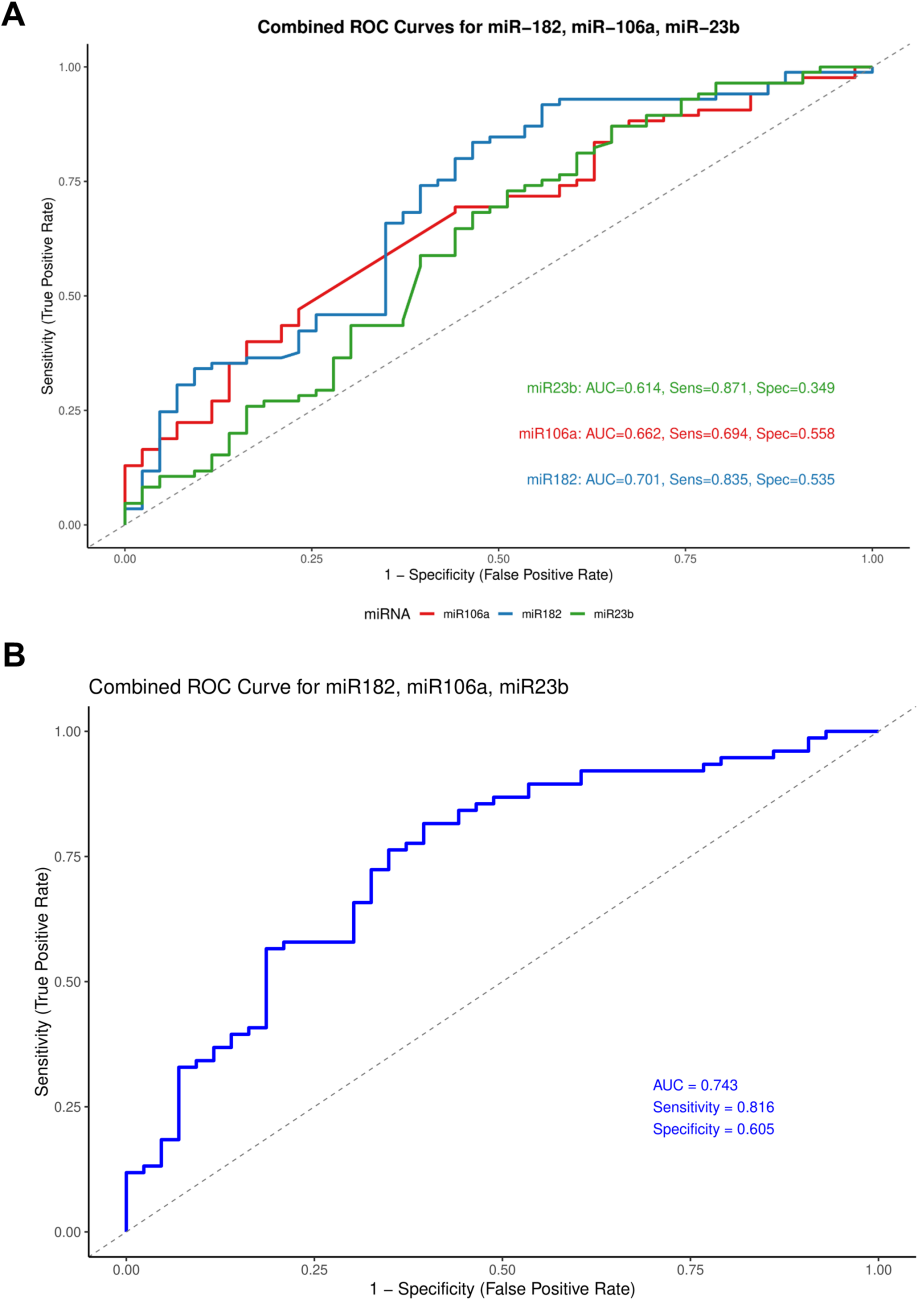
Receiver operating characteristic (ROC) curves for miR-182, miR-106a, and miR-23b. **(A)** Individual ROC curves representing the diagnostic performance of each miRNA. **(B)** Combined ROC curve comparing all three miRNAs on a single plot.

Receiver operating characteristic (ROC) curve analysis was performed to assess the diagnostic utility of the selected miRNAs. Among the ten miRNAs included in this study, miR-182, miR-106a, and miR-23b showed significant diagnostic performance with AUC values of 0.701, 0.662, and 0.614, respectively (**Figure 5A**). To evaluate whether a combination of these miRNAs could enhance diagnostic accuracy, we constructed a composite ROC curve using miR-182, miR-106a, and miR-23b. This combined panel showed improved diagnostic performance, yielding an AUC of 0.743 with a sensitivity of 81.6% and specificity of 60.5% (**Figure 5B**).

### 3.6 Multivariate Cox proportional hazards analysis

We performed a multivariate Cox proportional hazards regression analysis to evaluate the association between miRNA expression and progression-free survival (PFS), adjusting for the clinicopathological covariates. For the serum levels of ten selected miRNAs and progression-free survival (PFS) in ovarian cancer patients, the model included 78 patients with 41 progression events. Among the miRNAs tested, miR-130a was significantly associated with shorter PFS (Hazard Ratio [HR] = 0.64, 95% CI: 0.46–0.90, *p* = 0.010). Additionally, miR-200a showed a trend toward significance (*p* = 0.060). Among clinical variables, FIGO stage III (HR = 3.70, 95% CI: 1.24–11.03, *p* = 0.019) and FIGO stage IV (HR = 34.38, 95% CI: 2.99–395.33, *p* = 0.005) were independently associated with worse PFS, compared to stage I. Statistically significant associations were not observed for age, CA-125 levels, lymph node metastasis, presence of metastatic cells in ascites, or chemotherapy response score. The model showed a concordance index of 0.729, indicating good discriminative ability (**Table 3**).

**Table 3:** Multivariate Cox proportional hazards model assessing the association of serum miRNA expression and clinical variables with progression-free survival (PFS) in ovarian cancer patients.

Variable	Hazard ratio (HR)	95% C.I.	P value	Significance
miR-130a	0.64	0.46 - 0.90	0.010	*
miR-182	0.95	0.78 - 1.14	0.564	ns
miR-433	0.90	0.77 - 1.05	0.179	ns
miR-145	1.11	0.88 - 1.40	0.377	ns
miR-9	0.98	0.86 - 1.11	0.695	ns
miR-20a	0.96	0.84 - 1.09	0.540	ns
miR-23b	1.04	0.94 - 1.16	0.423	ns
miR-200a	1.50	0.98 - 2.29	0.060	ns
miR-365	1.08	0.93 - 1.26	0.325	ns
miR-106a	0.99	0.92 - 1.07	0.803	ns
Age	1.02	0.98 - 1.06	0.270	ns
Treatmentmode: Adjuvantvs. Neo-adjuvant	0.50	0.15 - 1.71	0.270	ns
FIGO-Stage 2 vs Stage 1	0.61	0.05 - 7.30	0.698	ns
FIGO-Stage 3 vs. Stage 1	3.70	1.24 - 11.03	0.019	*
FIGO-Stage 4 vs. Stage 1	34.38	2.99 - 395.33	0.005	**
CA-125 levels	1.00	0.99 - 1.00	0.840	ns
Lymph node metastasis	1.20	0.48 - 3.01	0.690	ns
Presence of metastatic cells in ascites	0.86	0.39 - 1.90	0.703	ns
Chemo response score (Good vs poor)	0.78	0.33 - 1.89	0.587	ns

*≤ 0.05, ** ≤ 0.01, ns- not significant.

Similarly, we evaluated the association of tissue miRNA expression and clinical variables with progression-free survival (PFS) in ovarian cancer patients using multivariate Cox proportional hazards modelling. The analysis included 65 patients, among whom 34 PFS events were observed. Model performance was acceptable, with a concordance index of 0.74, indicating good discriminative ability. In this model, only miR-20a expression in tumor tissue was significantly associated with shorter PFS (Hazard Ratio [HR] = 1.13, 95% CI: 1.01–1.28, *p* = 0.040). None of the other nine miRNAs showed statistically significant associations with PFS. Among clinical variables, FIGO stage IV demonstrated a strong trend toward significance (HR = 6.81, 95% CI: 0.95–48.56, *p* = 0.056), as did lymph node metastasis (HR = 2.28, 95% CI: 0.95–5.48, *p* = 0.066), and miR-106a (HR = 1.15, 95% CI: 0.98–1.35, *p* = 0.097). Other variables, including age, CA-125 levels, treatment mode, and presence of metastatic cells in ascites, were not significantly associated with PFS in this model (**Table 4**).

**Table 4:** Multivariate Cox proportional hazards model evaluating the association of tissue miRNA expression and clinical variables with progression-free survival (PFS) in ovarian cancer patients.

Variable	Hazard ratio (HR)	95% C.I.	P value	Significance
miR-130a	1.07	0.89 - 1.27	0.474	ns
miR-182	0.82	0.65 - 1.05	0.110	ns
miR-433	0.96	0.79 - 1.18	0.694	ns
miR-145	0.98	0.88 - 1.10	0.740	ns
miR-9	0.98	0.92 - 1.05	0.649	ns
miR-20a	1.13	1.01 - 1.28	0.040	*
miR-23b	0.98	0.88 - 1.08	0.666	ns
miR-200a	1.05	0.96 - 1.15	0.284	ns
miR-365	0.96	0.87 - 1.06	0.434	ns
miR-106a	1.15	0.98 - 1.35	0.097	ns
AGE	1.01	0.96 - 1.05	0.793	ns
Treatment mode: Adjuvantvs. Neo-adjuvant	1.69	0.63 - 4.55	0.297	ns
FIGO Stage 2 vs Stage 1	1.015	0.090 – 11.405	0.9907	ns
FIGO-Stage 3 vs. Stage 1	2.33	0.80 - 6.83	0.123	ns
FIGO-Stage 4 vs. Stage 1	6.81	0.95 - 48.56	0.056	ns
CA-125 levels	1.00	1.00 - 1.00	0.572	ns
Lymph node metastasis	2.28	0.95 - 5.48	0.066	ns
Presence of metastatic cells in ascites	1.16	0.50 - 2.69	0.736	ns
Chemo response score (Good vs poor)	1.71	0.59 - 4.92	0.319	ns

*≤ 0.05, ns- not significant.

## 4 Discussion

Ovarian cancer is a complicated disease with distinct molecular and biological characteristics. Despite of the successful treatment with first-line chemotherapy, a substantial number of patients develop recurrence of the disease and chemoresistance. miRNAs are key players in the development of chemoresistance. These small non-coding RNAs can be actively secreted into the circulation by tumor cells, and their levels tend to change with a reduction in tumor burden after surgical resection or after chemotherapeutic treatment, making them potential biomarkers for evaluating disease progression (27). Also, chemotherapy can influence miRNA export through several mechanisms, such as KRAS-dependent miRNA sorting into exosomes (13). Chemotherapeutic agents may disrupt this process by altering KRAS signaling, leading to changes in exosomal miRNA composition. These alterations may shift the circulating miRNA profile.

In our study, we observed altered serum miRNA expression patterns in women diagnosed with OC. Specifically, oncogenic miRNAs such as miR-182, miR-130a, miR-106a, and miR-200a were downregulated, while tumor suppressor miRNAs, including miR-433, miR-145, and miR-20a, were upregulated. These results are indicative of a tumor driven mechanism in which cells selectively release tumor suppressor miRNAs to promote oncogenic growth. Similar observation has been reported in non-small cell lung cancer, where oncogenic miRNAs are preferentially retained within tumor cells, and their release is suppressed during tumorigenesis (28). The selective release of miRNAs may occur through several mechanisms, including exosomal transport, where miRNAs are packaged into vesicles and secreted from the cell; active export mediated by RNA-binding proteins such as Argonaute 2 (Ago2); or passive release from tumor cells undergoing stress, damage, or cell death (29, 30).

The diagnostic potential of circulating miRNAs in ovarian cancer has gathered increasing interest due to their stability and detectability in body fluids. In our Receiver Operating Curve analysis (ROC), miR-182, miR-106a, and miR-23b demonstrated moderate individual diagnostic accuracy, with miR-182 yielding the highest individual discriminatory power (AUC = 0.701). Diagnostic performance improved when the three miRNAs were combined, achieving an AUC of 0.743 with a sensitivity of 81.6% and specificity of 60.5%. However, the moderate specificity highlights the need for further refinement and validation in larger cohorts.

miR-182 is of particular interest, as it is one of the eight miRNAs that controlled 89% of the miRNA-associated genes of the mesenchymal subtype in the TCGA (The Cancer Genome Atlas) data (31). Increased levels of miR-182 have been observed in several ovarian cancer cell lines compared to normal ovarian surface epithelium (32). Similarly, elevated expression of miR-182 in ovarian cancer (OC) tissues, relative to normal ovarian tissue, has been reported to promote cell proliferation, invasion, and chemoresistance (33). While elevated miR-9 expression in the tissues promotes epithelial-to-mesenchymal transition in OC (26). The reduced expression of miR-182 and miR-9 observed in chemo treated patients in our study may therefore reflect a treatment-induced suppression of these oncogenic miRNAs.

Further, we assessed the expression of these oncogenic miRNAs in relation to key clinical variables, including disease stage, presence of metastasis in ascitic or peritoneal fluid, timing of relapse (early or late), and chemotherapy response score, in both tumor tissues and serum samples. The survival rate of OC is about 93% for women diagnosed at an early stage, which drops to 30% upon late diagnosis (34). In our study, around 63% of patients were diagnosed at an advanced stage. These advanced-stage patients had comparatively higher levels of oncogenic miR-182, miR-20a, and miR-106a in the tumor tissues. Elevated miR-106a expression can modulate proliferation and is associated with paclitaxel resistance in OC (20). Whereas overexpression of miR-182 targets genes involved in DNA repair and is associated with shorter overall survival of OC patients (35, 36). Elevated expression of miR-20a in tumor tissues of advanced-stage ovarian cancer patients has been shown to reduce the cytotoxic activity of natural killer (NK) cells by downregulating MICA/B expression (37). Although MICA/B levels are generally higher in ovarian cancer tissues compared to normal ovarian epithelium, their expression does not appear to change significantly in relapsed patients (38, 39). In our study, miR-20a expression in tumor tissues was significantly associated with shorter progression-free survival in the multivariate cox proportional hazards model, supporting previous studies implicating miR-20a in promoting oncogenic processes such as proliferation and chemoresistance (40, 41).

Additionally, we found elevated levels of oncogenic miRNAs like miR-182 and miR-200a in the serum of OC patients with the presence of metastatic cells in their ascites. Two studies support this finding as the overexpression of miR-200a in the serum is associated with metastasis, advanced stage, and progression of OC (42, 43). Conversely, the levels of tumor-suppressive miRNAs like miR-433 and miR-145 were significantly downregulated in the tissue of patients with the presence of lymph node metastasis. Downregulation of miR-433 inhibits its tumor-suppressive role and is associated with proliferation, metastasis and invasion in multiple cancers (21). Similarly, miR-145 is a key player in the regulation of cell migration and invasion in multiple cancers including OC (22).

Due to delayed diagnosis of OC, most of the patients are presented with metastasized disease making it challenging to achieve complete resection of the tumor. Hence, the effectiveness of the chemotherapeutic regimen plays a major role in the prognosis of OC. In our study, we report that the expression levels of oncogenic miRNAs like miR-182 and miR-130a were higher in the serum of patients with better chemo response score, potentially reflecting chemotherapy-induced release of these miRNAs from tumor tissue. miR-130a has been implicated in the pathogenesis of many cancers including OC and plays a vital role in the development of chemoresistance by acting as an intermediary in pathways such as Wnt/β-catenin, PI3K/Akt/PTEN/mTOR and NF-κB/PTEN (44). Notably, serum miR-130a was significantly associated with improved PFS, suggesting a potential tumor-suppressive role in the circulating compartment, consistent with its reported involvement in cell cycle regulation and apoptosis (44, 45).Overexpression of miR-182 is associated with poor prognosis of bladder, colorectal, and hepatocellular cancers (46–48). While higher expression of the miR-106 family was related to poor survival of colorectal cancer patients. We report that the expression of oncogenic miRNAs, miR-182, miR-106a, and miR-200a were elevated in the serum of patients with early relapse compared to those with late relapse. Lower miR-9 expression is associated with shorter survival of EOC (49). In our study also, lower miR-9 levels were evident in patients with an early relapse.

The expression levels of the miRNAs studied were not significantly associated with the overall survival of OC patients. The comparatively brief follow-up period may have impeded the prognostic studies. Other limitations of the study were the unavailability of adjacent healthy ovarian tissues (due to ethical issues) for comparison with tumor tissues and small sample sizes in certain sub groups like chemo response score and relapse, which may have limited statistical power. However, the merits of the study include a substantial patient cohort of a single subtype of OC and the availability of paired serum and tissue specimens. The study also highlights the effect of chemotherapy on the expression levels of both oncomiRs and tumor suppressor miRNAs and their probable roles in predicting the prognosis of the disease in OC patients from India. Possibly for the first time in an Indian cohort, this study also reports the expression of these miRNAs both in tumor tissues and serum. It examines their correlation with clinical parameters such as stage of the disease, presence of metastasis in ascites or peritoneal fluid, early or late relapse of the disease, and chemo response score, indicating their possible prognostic value in OC treatment. Notably, Indian OC patients may exhibit unique miRNA expression patterns compared to global cohorts. These differences could arise from the considerable genetic heterogeneity within the Indian population (50), along with distinct environmental, dietary, and lifestyle influences that differ from those in Western countries. Additionally, variations in tumor biology, clinical presentation, and epigenetic regulation may contribute to these distinct profiles (10). These findings emphasize the importance of region-specific studies to improve our understanding of the disease and enhance the clinical relevance of miRNA-based studies in diverse populations.

### 4.1 Conclusion

In conclusion, the study reiterates that the rate of early diagnosis of OC is very poor in the urban Indian population. We report the phenomenon of selective release of tumor suppressive miRs while retaining the oncogenic miRs within tumor cells to achieve higher proliferation rates. Elevated serum levels of oncogenic miR-182 and miR-130a were associated with chemotherapy response, suggesting their potential as predictive biomarkers. Similarly, elevated levels of miR-106a in patients with poor chemo response and early relapse suggest its role as an oncogenic miRNA. Our data also highlights the significance of miR-200a associated with the metastasis and poor prognosis of OC. Additionally, a combined panel of miR-182, miR-106a, and miR-23b demonstrated enhanced diagnostic performance, supporting their clinical utility. The expression of miR-130a in serum and miR-20a in tumor tissue emerged as independent predictors of progression free survival, underlining their potential as prognostic biomarkers. These observations emphasize the importance of region-specific biomarker studies and provide a foundation for further validation of these miRNAs in a larger cohort with longer follow-up periods to better understand their prognostic and predictive roles in ovarian cancer management.

## Data Availability

The original contributions presented in the study are included in the article/**Supplementary Material**. Further inquiries can be directed to the corresponding author.

## Supplementary material

Supplementary Figure 1
**(A)** Relative levels of miRNAs in serum of patients with ovarian cancer and healthy. Relative levels of **(B)** miR-200a, **(C)** miR-106a, **(D)** miR-9, **(E)** miR-23b, **(F)** miR-433, and **(G)** miR-145 in sequential serum samples collected from ovarian cancer patients (n = 16) at chemo-naive and each consecutive chemotherapy cycle follow-up time point. (Data are presented as Mean ± SEM) * p < 0.05.

Supplementary Figure 2 Relative expression levels of **(A)** miR-365 in paired serum samples collected from ovarian cancer patients (n = 16) at chemo-naive and consecutive chemotherapy cycle follow-up time points. **(B)** The relative miRNA levels in tumor tissues of chemo-naive and chemotherapy-treated ovarian cancer patients. **(C)** The difference between miRNA levels in tumor tissue of patients based on FIGO stage of the disease. (Data are presented as Mean ± SEM) * p < 0.05.

Supplementary Figure 3 Comparison of relative miRNA levels in ovarian cancer patients grouped based on the presence or absence of metastatic cells in their ascitic fluid. **(A)** Serum miRNA levels **(B)** Tissue miRNA levels. (Data are presented as Mean ± SEM) * p < 0.05.

Supplementary Figure 4 Comparison of relative miRNA levels in ovarian cancer patients grouped based on the presence or absence of lymph node metastasis. **(A)** Serum miRNA levels **(B)** Tissue miRNA levels. (Data are presented as Mean ± SEM) * p < 0.05.

Supplementary Figure 5 Comparison of miRNA expression levels in patients divided into two groups based on chemo response score **(A)** serum levels, **(B)** tissue levels. (Data are presented as Mean ± SEM) * p < 0.05.

Supplementary Figure 6 Comparison of miRNA expression levels in two groups of ovarian cancer patients in the **(A)** serum, and **(B)** tissue specimens based on early or late relapse of the disease. (Data are presented as Mean ± SEM) * p < 0.05.

Supplementary Figure 7 Relative fold change in the expression of serum miRNAs in chemonaive and chemotreated ovarian cancer patients in comparison to women enrolled as healthy controls. (Data are presented as Mean ± SEM) * p < 0.05.

Supplementary Figure 8 Receiver Operating Characteristics (ROC) curves for **(A)** miR-130a, **(B)** miR-433, **(C)** miR-145, **(D)** miR-9, **(E)** miR-20a, **(F)** miR-200a, and **(G)** miR-365. * p < 0.05.

Supplementary Figure 9 Kaplan Meier curves displaying the association of **(A)** mode of treatment, **(B)** FIGO Stage of the disease, **(C)** serum levels of CA-125 and **(D)** presence or absence of lymph node metastasis in ovarian cancer patients and their respective overall survival. * p < 0.05.

## References

[1] Bray F Ferlay J Soerjomataram I Siegel RL Torre LA Jemal A. Global cancer statistics 2018: GLOBOCAN estimates of incidence and mortality worldwide for 36 cancers in 185 countries. CA Cancer J Clin. (2018) 68:394–424. doi: 10.3322/caac.21492, PMID: 30207593

[2] du Bois A Reuss A Pujade-Lauraine E Harter P Ray-Coquard I Pfisterer J. Role of surgical outcome as prognostic factor in advanced epithelial ovarian cancer: a combined exploratory analysis of 3 prospectively randomized phase 3 multicenter trials: by the ArbeitsgemeinschaftGynaekologischeOnkologieStudiengruppeOvarialkarzi. Cancer. (2009) 115:1234–44. doi: 10.1002/cncr.24149, PMID: 19189349

[3] Wright AA Bohlke K Armstrong DK Bookman MA Cliby WA Coleman RL. Neoadjuvant chemotherapy for newly diagnosed, advanced ovarian cancer: Society of Gynecologic Oncology and American Society of Clinical Oncology Clinical Practice Guideline. Gynecol Oncol. (2016) 143:3–15. doi: 10.1016/j.ygyno.2016.05.022, PMID: 27650684 PMC5413203

[4] Kumar P Ranmale S Tongaonkar H Mania-Pramanik J. Immune profile of blood, tissue and peritoneal fluid: A comparative study in high grade serous epithelial ovarian cancer patients at interval debulking surgery. Vaccines (Basel). (2022) 10(12):2121. doi: 10.3390/vaccines10122121, PMID: 36560531 PMC9784879

[5] Allemani C Matsuda T Di Carlo V Harewood R Matz M Nikšić M. Global surveillance of trends in cancer survival 2000–14 (CONCORD-3): analysis of individual records for 37 513 025 patients diagnosed with one of 18 cancers from 322 population-based registries in 71 countries. Lancet. (2018) 391:1023–75. doi: 10.1016/S0140-6736(17)33326-3, PMID: 29395269 PMC5879496

[6] Pokhriyal R Hariprasad R Kumar L Hariprasad G. Chemotherapy resistance in advanced ovarian cancer patients. biomark Cancer. (2019) 11:1179299X1986081. doi: 10.1177/1179299X19860815, PMID: 31308780 PMC6613062

[7] Deb B Uddin A Chakraborty S. miRNAs and ovarian cancer: An overview. J Cell Physiol. (2018) 233:3846–54. doi: 10.1002/jcp.26095, PMID: 28703277

[8] Nakamura K Sawada K Yoshimura A Kinose Y Nakatsuka E Kimura T. Clinical relevance of circulating cell-free microRNAs in ovarian cancer. Mol Cancer. (2016) 15:48. doi: 10.1186/s12943-016-0536-0, PMID: 27343009 PMC4921011

[9] Iorio MV Visone R Di Leva G Donati V Petrocca F Casalini P. MicroRNA signatures in human ovarian cancer. Cancer Res. (2007) 67:8699–707. doi: 10.1158/0008-5472.CAN-07-1936, PMID: 17875710

[10] Kumar V Gupta S Chaurasia A Sachan M. Evaluation of diagnostic potential of epigenetically deregulated miRNAs in epithelial ovarian cancer. Front Oncol. (2021) 11. doi: 10.3389/fonc.2021.681872, PMID: 34692473 PMC8529058

[11] Kanlikilicer P Rashed MH Bayraktar R Mitra R Ivan C Aslan B. Ubiquitous release of exosomal tumor suppressor miR-6126 from ovarian cancer cells. (2016) 76(24):7194–207. doi: 10.1158/0008-5472.CAN-16-0714, PMID: 27742688 PMC5901763

[12] Zou X Zhao Y Liang X Wang H Zhu Y Shao Q. Double insurance for OC: miRNA-mediated platinum resistance and immune escape. Front Immunol. (2021) 12:1–14. doi: 10.3389/fimmu.2021.641937, PMID: 33868274 PMC8047328

[13] Cha DJ Franklin JL Dou Y Liu Q Higginbotham JN Beckler MD. KRAS-dependent sorting of miRNA to exosomes. Elife. (2015) 4:e07197. doi: 10.7554/eLife.07197.027, PMID: 26132860 PMC4510696

[14] Bolukbasi MF Mizrak A Ozdener GB Madlener S Ströbel T Erkan EP. miR-1289 and “Zipcode”-like sequence enrich mRNAs in microvesicles. Mol Ther Nucleic Acids. (2012) 1:e10. doi: 10.1038/mtna.2011.2, PMID: 23344721 PMC3381601

[15] Koppers-Lalic D Hackenberg M Bijnsdorp IV van Eijndhoven MAJ Sadek P Sie D. Nontemplated nucleotide additions distinguish the small RNA composition in cells from exosomes. Cell Rep. (2014) 8:1649–58. doi: 10.1016/j.celrep.2014.08.027, PMID: 25242326

[16] Hummel R Hussey DJ Haier J. MicroRNAs: Predictors and modifiers of chemo- and radiotherapy in different tumour types. Eur J Cancer. (2010). doi: 10.1016/j.ejca.2009.10.027, PMID: 19948396

[17] Komatsu S Kiuchi J Imamura T Ichikawa D Otsuji E. Circulating microRNAs as a liquid biopsy: a next-generation clinical biomarker for diagnosis of gastric cancer. J Cancer Metastasis Treat. (2018) 4:36. doi: 10.20517/2394-4722.2017.58

[18] Kumar V Gupta S Varma K Chaurasia A Sachan M. Diagnostic performance of microRNA-34a, let-7f and microRNA-31 in epithelial ovarian cancer prediction. J Gynecol Oncol. (2022) 33(4):e49. doi: 10.3802/jgo.2022.33.e49, PMID: 35557032 PMC9250857

[19] Li N Yang L Wang H Yi T Jia X Chen C. MiR-130a and MiR-374a function as novel regulators of cisplatin resistance in human ovarian cancer A2780 cells. PloS One. (2015) 10:1–12. doi: 10.1371/journal.pone.0128886, PMID: 26043084 PMC4456206

[20] Huh JH Kim TH Kim K Song JA Jung YJ Jeong JY. Dysregulation of miR-106a and miR-591 confers paclitaxel resistance to ovarian cancer. Br J Cancer. (2013) 109:452–61. doi: 10.1038/bjc.2013.305, PMID: 23807165 PMC3721386

[21] Tang J Chen J Wang Y Zhou S. The role of MiRNA-433 in Malignant tumors of digestive tract as tumor suppressor. Cancer Rep. (2022) 5(9):e1694. doi: 10.1002/cnr2.1694, PMID: 35976177 PMC9458491

[22] Xu WX Liu Z Deng F Wang DD Li XW Tian T. MiR-145: a potential biomarker of cancer migration and invasion. Am J Transl Res. (2019) 11:6739–53. doi: 10.1002/cnr2.1694, PMID: 31814885 PMC6895535

[23] Zhang Q Wang Q Sun W Gao F Liu L Cheng L. Change of circulating and tissue-based miR-20a in human cancers and associated prognostic implication: A systematic review and meta-analysis. BioMed Res Int. (2018) 2018:6124927. doi: 10.1155/2018/6124927, PMID: 30596096 PMC6286746

[24] Su L Liu M. Correlation analysis on the expression levels of microRNA-23a and microRNA-23b and the incidence and prognosis of ovarian cancer. Oncol Lett. (2018) 16:262–6. doi: 10.3892/ol.2018.8669, PMID: 29928410 PMC6006491

[25] Choi PW Ng SW. The functions of MicroRNA-200 family in ovarian cancer: Beyond Epithelial-Mesenchymal transition. Int J Mol Sci. (2017) 18(6):1207. doi: 10.3390/ijms18061207, PMID: 28587302 PMC5486030

[26] Zhou B Xu H Xia M Sun C Li N Guo E. Overexpressed miR-9 promotes tumor metastasis via targeting E-cadherin in serous ovarian cancer. Front Med. (2017) 11:214–22. doi: 10.1007/s11684-017-0518-7, PMID: 28470508

[27] Condrat CE Thompson DC Barbu MG Bugnar OL Boboc A Cretoiu D. miRNAs as biomarkers in disease: latest findings regarding their role in diagnosis and prognosis. Cells. (2020) 9:1–32. doi: 10.3390/cells9020276, PMID: 31979244 PMC7072450

[28] Aiso T Ohtsuka K Ueda M Karita S Yokoyama T Takata S. Serum levels of candidate microRNA diagnostic markers differ among the stages of non-small-cell lung cancer. Oncol Lett. (2018) 16:6643–51. doi: 10.3892/ol.2018.9464, PMID: 30405804 PMC6202492

[29] Valadi H Ekström K Bossios A Sjöstrand M Lee JJ Lötvall JO. Exosome-mediated transfer of mRNAs and microRNAs is a novel mechanism of genetic exchange between cells. Nat Cell Biol. (2007) 9(6):654–9. doi: 10.1038/ncb1596, PMID: 17486113

[30] Mitchell PS Parkin RK Kroh EM Fritz BR Wyman SK Pogosova-Agadjanyan EL. Circulating microRNAs as stable blood-based markers for cancer detection. Proc Natl Acad Sci. (2008) 105(30):10513–8. 10.1073/pnas.0804549105 PMC2492472 18663219

[31] Yang D Sun Y Hu L Zheng H Ji P Pecot CV. Integrated analyses identify a master microRNA regulatory network for the mesenchymal subtype in serous ovarian cancer. Cancer Cell. (2013) 23:186–99. doi: 10.1016/j.ccr.2012.12.020, PMID: 23410973 PMC3603369

[32] Kan CWS Hahn MA Gard GB Maidens J Huh JY Marsh DJ. Elevated levels of circulating microRNA-200 family members correlate with serous epithelial ovarian cancer. BMC Cancer. (2012) 12:627. doi: 10.1186/1471-2407-12-627, PMID: 23272653 PMC3542279

[33] Wang L Zhu MJ Ren AM Wu HF Han WM Tan RY. A ten-microRNA signature identified from a genome-wide microRNA expression profiling in human epithelial ovarian cancer. PloS One. (2014) 9:e96472. doi: 10.1371/journal.pone.0096472, PMID: 24816756 PMC4015980

[34] Hurwitz LM Pinsky PF Trabert B. General population screening for ovarian cancer. Lancet. (2021) 397:2128–30. doi: 10.1016/S0140-6736(21)01061-8, PMID: 33991478

[35] Marzec-Kotarska B Cybulski M Kotarski JC Ronowicz A Tarkowski R Polak G. Molecular bases of aberrant miR-182 expression in ovarian cancer. Genes Chromosomes Cancer. (2016) 55:877–89. doi: 10.1002/gcc.22387, PMID: 27295517

[36] Krishnan K Steptoe AL Martin HC Wani S Nones K Waddell N. MicroRNA-182-5p targets a network of genes involved in DNA repair. Rna. (2013) 19:230–42. doi: 10.1261/rna.034926.112, PMID: 23249749 PMC3543090

[37] Xie J Liu M Li Y Nie Y Mi Q Zhao S. Ovarian tumor-associated microRNA-20a decreases natural killer cell cytotoxicity by downregulating MICA/B expression. Cell Mol Immunol. (2014) 11:495–502. doi: 10.1038/cmi.2014.30, PMID: 24813230 PMC4197204

[38] Ghadially H Brown L Lloyd C Lewis L Lewis A Dillon J. MHC class i chain-related protein A and B (MICA and MICB) are predominantly expressed intracellularly in tumour and normal tissue. Br J Cancer. (2017) 116:1208–17. doi: 10.1038/bjc.2017.79, PMID: 28334733 PMC5418453

[39] Kumar P Ranmale S Mehta S Tongaonkar H Patel V Singh AK. Immune profile of primary and recurrent epithelial ovarian cancer cases indicates immune suppression, a major cause of progression and relapse of ovarian cancer. J Ovarian Res. (2023) 16(1):114. doi: 10.1186/s13048-023-01192-4, PMID: 37322531 PMC10268537

[40] Fan X Liu Y Jiang J Ma Z Wu H Liu T. MiR-20a promotes proliferation and invasion by targeting APP in human ovarian cancer cells. Acta BiochimBiophys Sin (Shanghai). (2010) 42:318–24. doi: 10.1093/abbs/gmq026, PMID: 20458444

[41] Liu Y Han S Li Y Liu Y Zhang D Li Y. MicroRNA-20a contributes to cisplatin-resistance and migration of OVCAR3 ovarian cancer cell line. Oncol Lett. (2017) 14:1780–6. doi: 10.3892/ol.2017.6348, PMID: 28789409 PMC5529943

[42] Zuberi M Mir R Das J Ahmad I Javid J Yadav P. Expression of serum miR-200a, miR-200b, and miR-200c as candidate biomarkers in epithelial ovarian cancer and their association with clinicopathological features. Clin Trans Oncol. (2015) 17:779–87. doi: 10.1007/s12094-015-1303-1, PMID: 26063644

[43] Meng X Müller V Milde-Langosch K Trillsch F Pantel K Schwarzenbach H. Diagnostic and prognostic relevance of circulating exosomal miR-373, miR-200a, miR-200b and miR-200c in patients with epithelial ovarian cancer. Oncotarget. (2016)., PMID: 26943577 10.18632/oncotarget.7850 PMC4941360

[44] Zhang H Jiang Lh Sun Dw Li J Ji Zl. The role of miR-130a in cancer. Breast Cancer. (2017) 24:521–7. doi: 10.1007/s12282-017-0776-x, PMID: 28477068

[45] Wei H Cui R Bahr J Zanesi N Luo Z Meng W. miR-130a deregulates PTEN and stimulates tumor growth. Cancer Res. (2017) 77:6168–79. doi: 10.1158/0008-5472.CAN-17-0530, PMID: 28935812 PMC7081380

[46] Chen H Xu L Wang L. Expression of miR-182 and Foxo3a in patients with bladder cancer correlate with prognosis. Int J Clin Exp Pathol. (2019) 12:4193–203. doi: 10.1186/1471-2407-12-627, PMID: 31933819 PMC6949789

[47] Liu H Du L Wen Z Yang Y Li J Wang L. Up-regulation of miR-182 expression in colorectal cancer tissues and its prognostic value. Int J Colorectal Dis. (2013) 28:697–703. doi: 10.1007/s00384-013-1674-0, PMID: 23474644

[48] Qin J Luo M Qian H Chen W. Upregulated miR-182 increases drug resistance in cisplatin-treated HCC cell by regulating TP53INP1. Gene. (2014) 538:342–7. doi: 10.1016/j.gene.2013.12.043, PMID: 24447717

[49] Li X Pan Q Wan X Mao Y Lu W Xie X. Methylation-associated Has-miR-9 deregulation in paclitaxel- resistant epithelial ovarian carcinoma. BMC Cancer. (2015) 15:509. doi: 10.1186/s12885-015-1509-1, PMID: 26152689 PMC4495847

[50] Jain A Bhoyar RC Pandhare K Mishra A Sharma D Imran M. IndiGenomes: A comprehensive resource of genetic variants from over 1000 Indian genomes. Nucleic Acids Res. (2021) 49:D1225–32. doi: 10.1093/nar/gkaa923., PMID: 33095885 PMC7778947

