# Supplementary figures and images for: Chemotherapy-induced alterations in miRNA expression and their prognostic implications in ovarian cancer
Source: Front Oncol. 2025 Aug 22;15:1580565. doi: 10.3389/fonc.2025.1580565 (PMC12411204; doi:10.3389/fonc.2025.1580565)

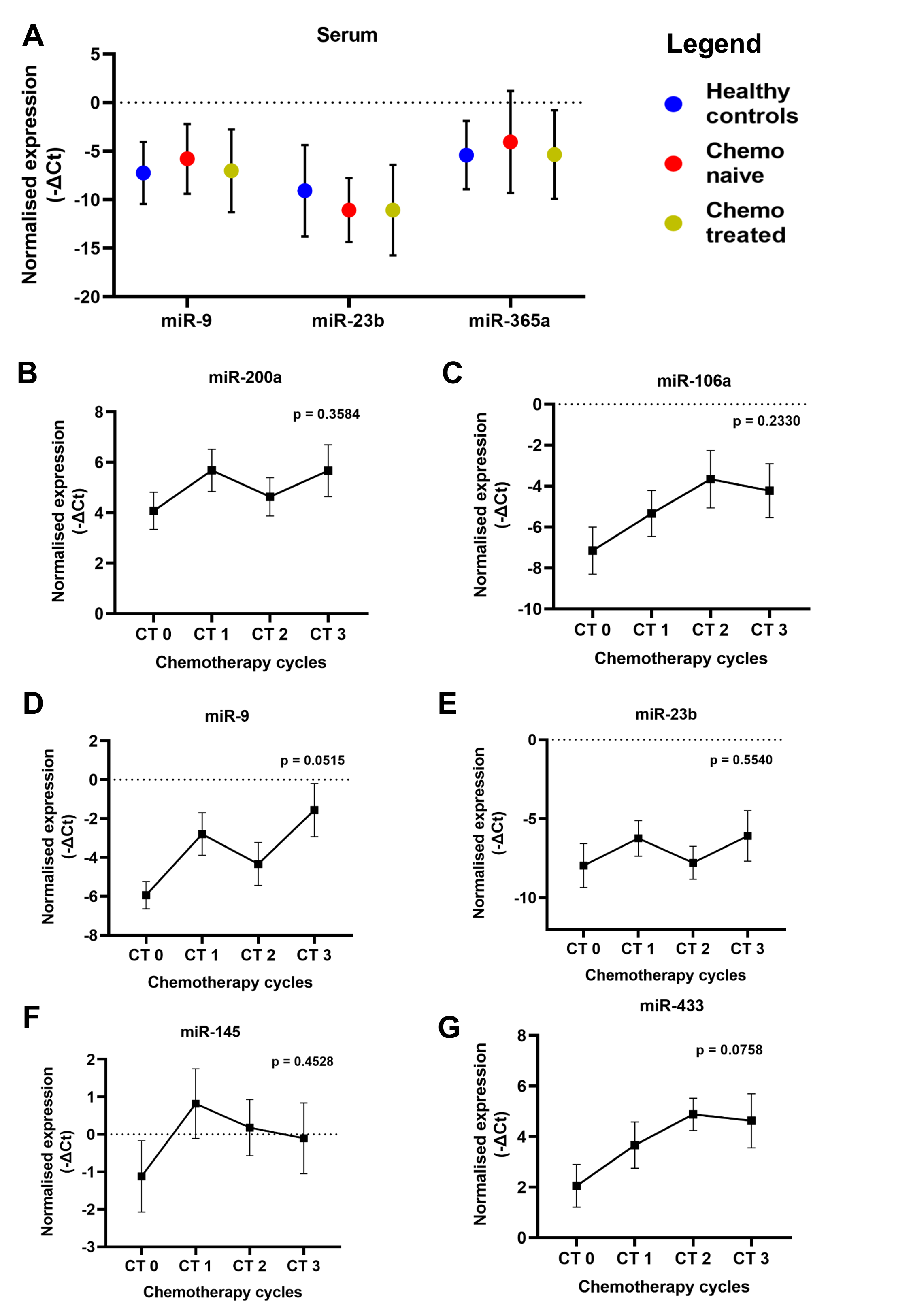

Supplement: Supplementary Figure 1 — (A) Relative levels of miRNAs in serum of patients with ovarian cancer and healthy. Relative levels of (B) miR-200a, (C) miR-106a, (D) miR-9, (E) miR-23b, (F) miR-433, and (G) miR-145 in sequential serum samples collected from ovarian cancer patients (n = 16) at chemo-naive and each consecutive chemotherapy cycle follow-up time point. (Data are presented as Mean ± SEM) * p < 0.05. [file Image1.tif]

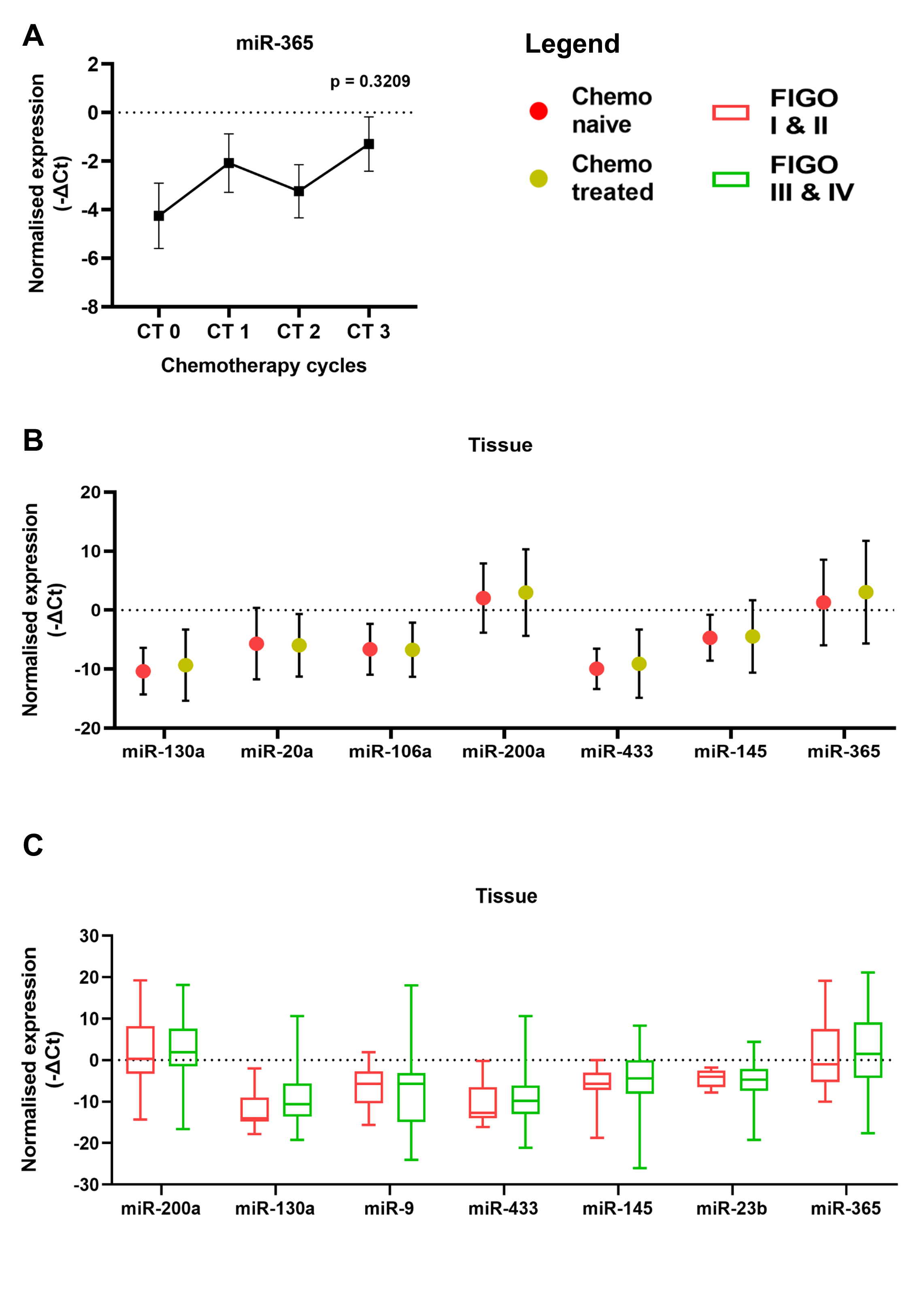

Supplement: Supplementary Figure 2 — Relative expression levels of (A) miR-365 in paired serum samples collected from ovarian cancer patients (n = 16) at chemo-naive and consecutive chemotherapy cycle follow-up time points. (B) The relative miRNA levels in tumor tissues of chemo-naive and chemotherapy-treated ovarian cancer patients. (C) The difference between miRNA levels in tumor tissue of patients based on FIGO stage of the disease. (Data are presented as Mean ± SEM) * p < 0.05. [file Image2.tif]

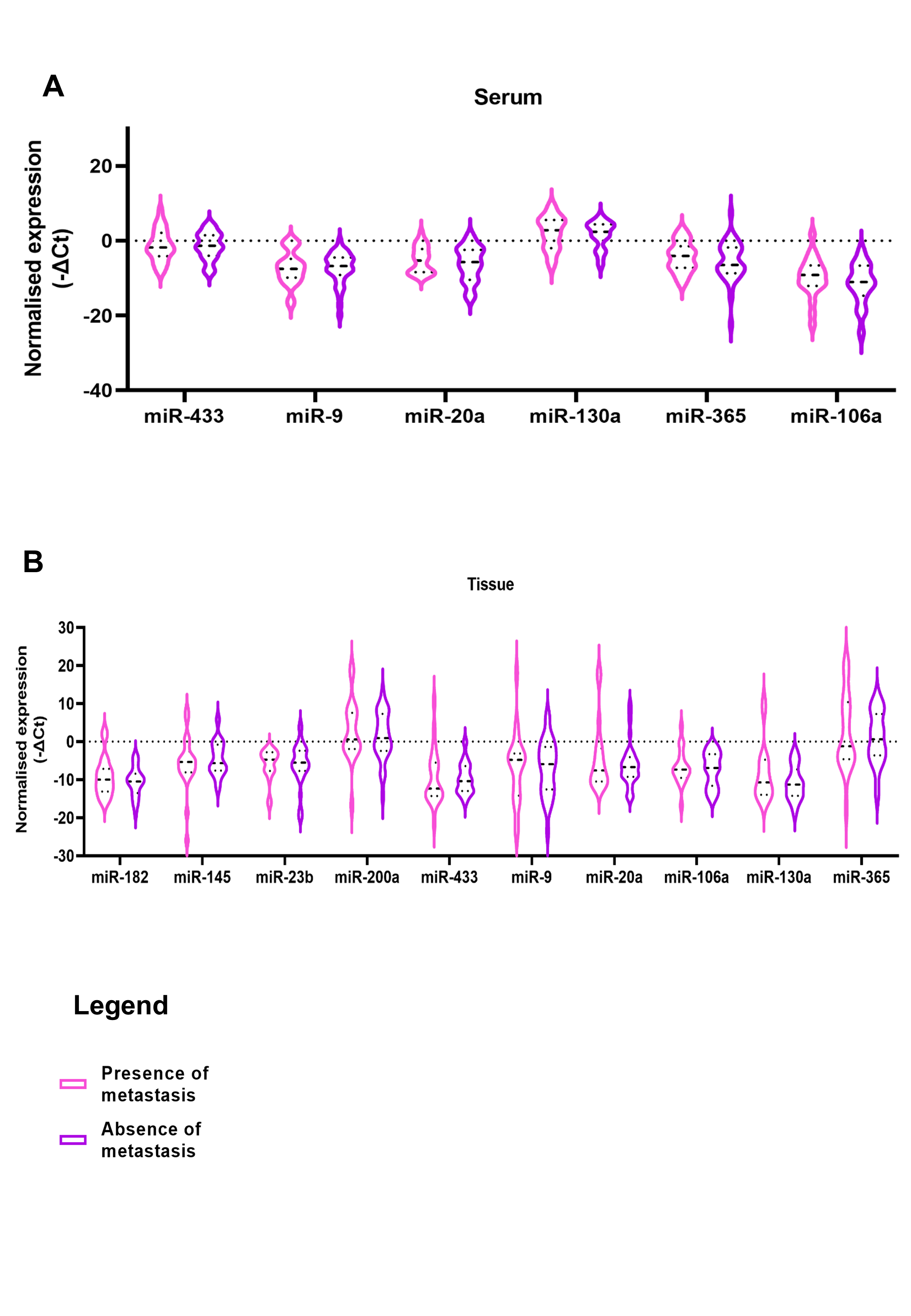

Supplement: Supplementary Figure 3 — Comparison of relative miRNA levels in ovarian cancer patients grouped based on the presence or absence of metastatic cells in their ascitic fluid. (A) Serum miRNA levels (B) Tissue miRNA levels. (Data are presented as Mean ± SEM) * p < 0.05. [file Image3.tif]

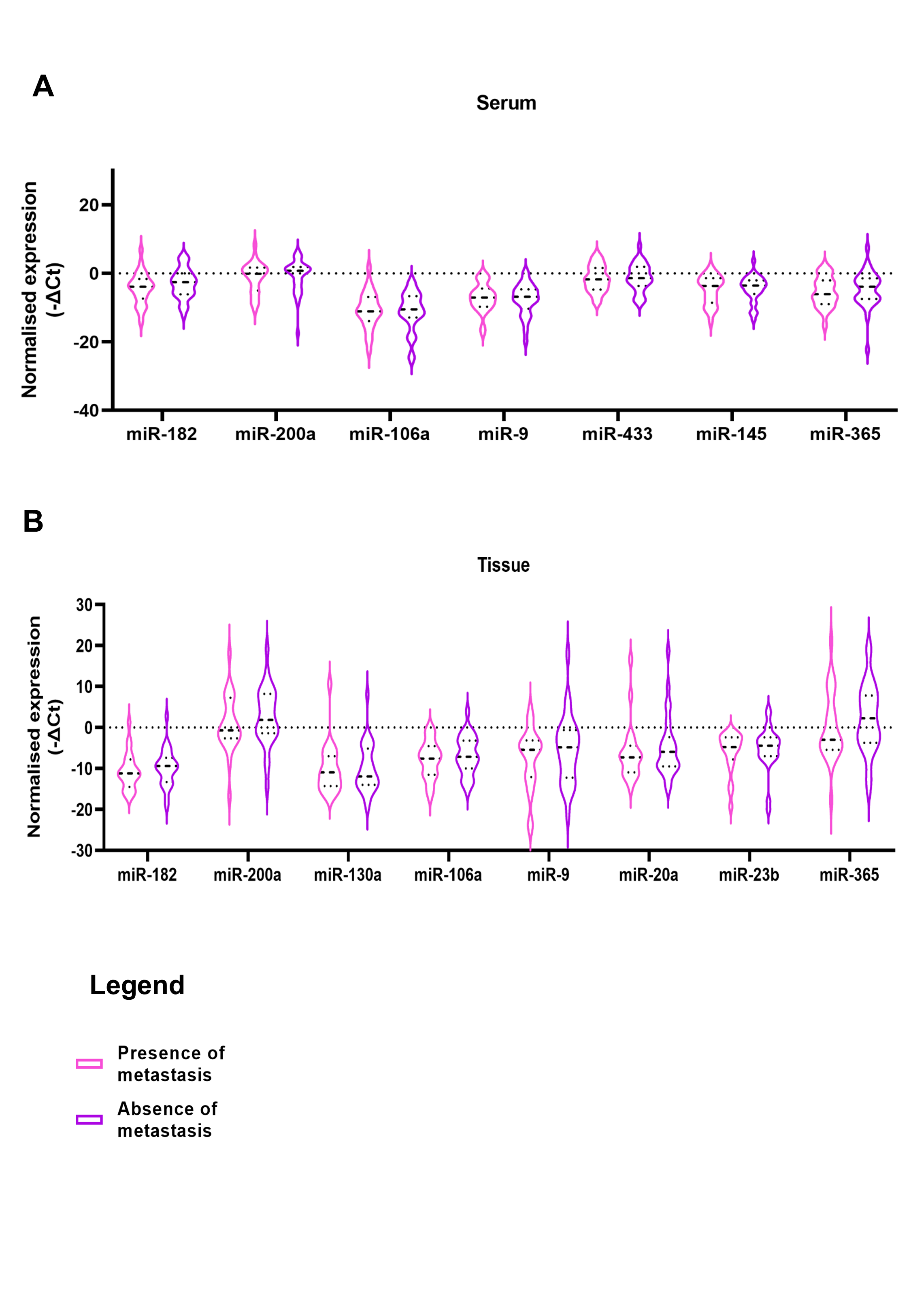

Supplement: Supplementary Figure 4 — Comparison of relative miRNA levels in ovarian cancer patients grouped based on the presence or absence of lymph node metastasis. (A) Serum miRNA levels (B) Tissue miRNA levels. (Data are presented as Mean ± SEM) * p < 0.05. [file Image4.tif]

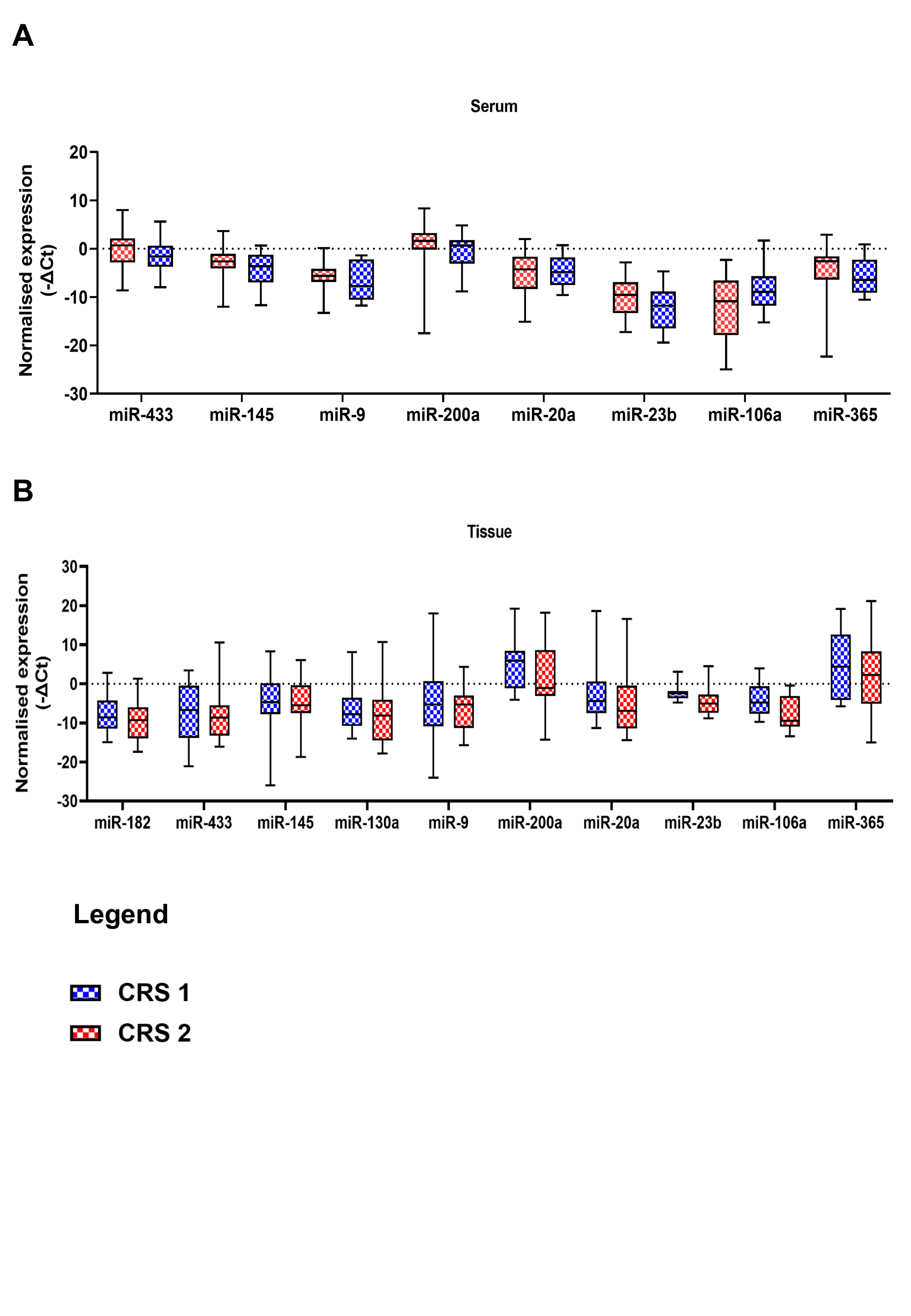

Supplement: Supplementary Figure 5 — Comparison of miRNA expression levels in patients divided into two groups based on chemo response score (A) serum levels, (B) tissue levels. (Data are presented as Mean ± SEM) * p < 0.05. [file Image5.tif]

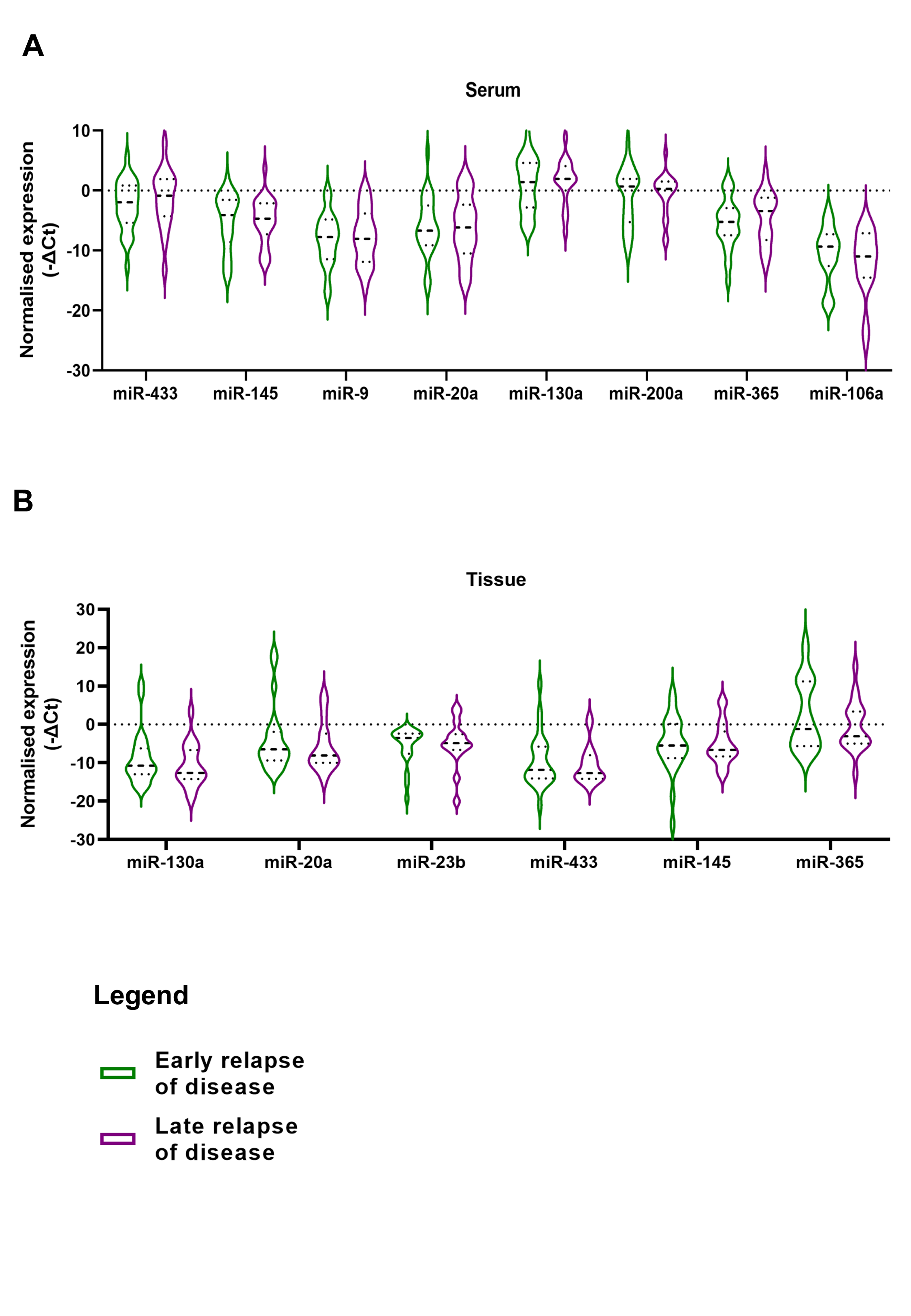

Supplement: Supplementary Figure 6 — Comparison of miRNA expression levels in two groups of ovarian cancer patients in the (A) serum, and (B) tissue specimens based on early or late relapse of the disease. (Data are presented as Mean ± SEM) * p < 0.05. [file Image6.tif]

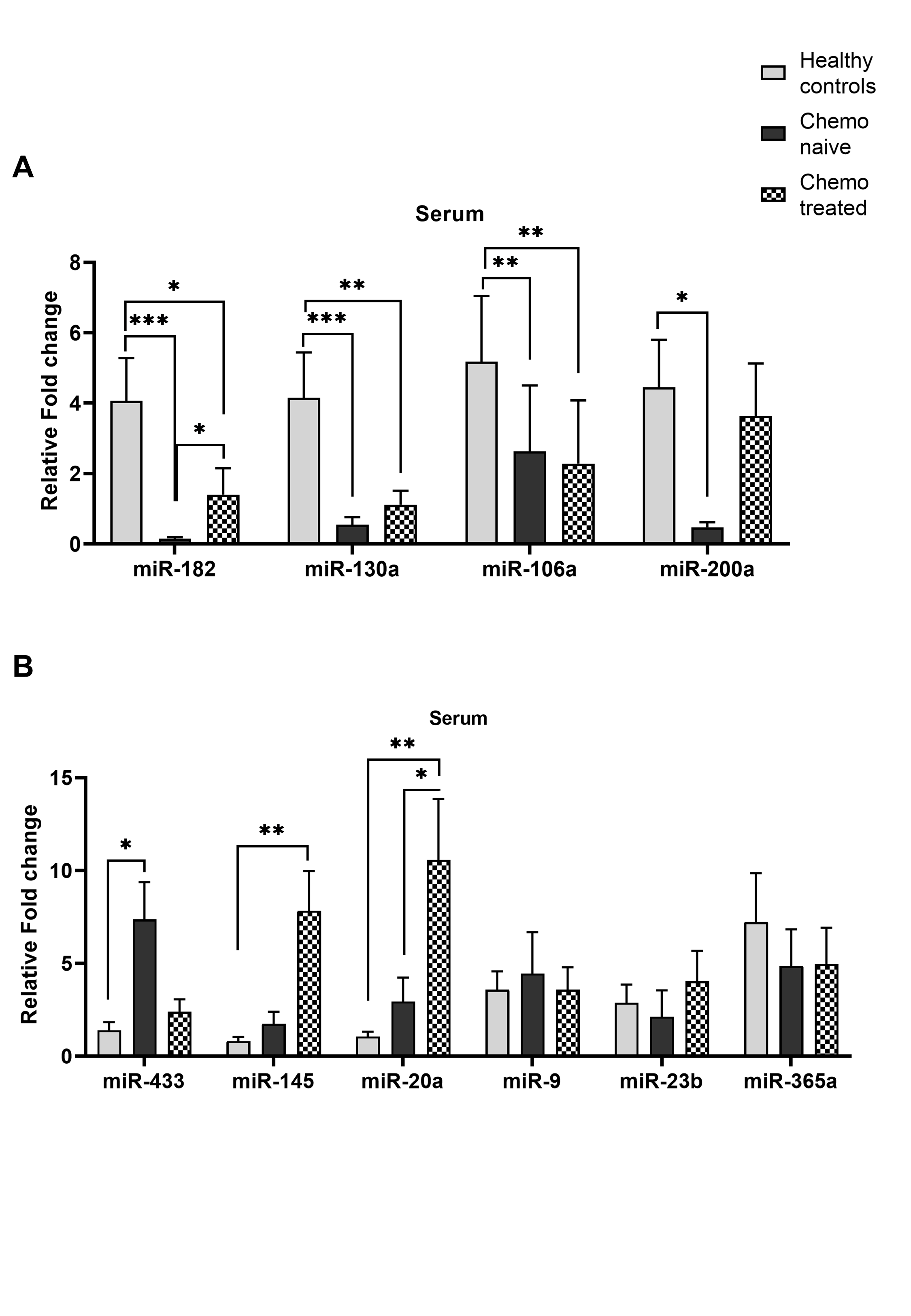

Supplement: Supplementary Figure 7 — Relative fold change in the expression of serum miRNAs in chemonaive and chemotreated ovarian cancer patients in comparison to women enrolled as healthy controls. (Data are presented as Mean ± SEM) * p < 0.05. [file Image7.tif]

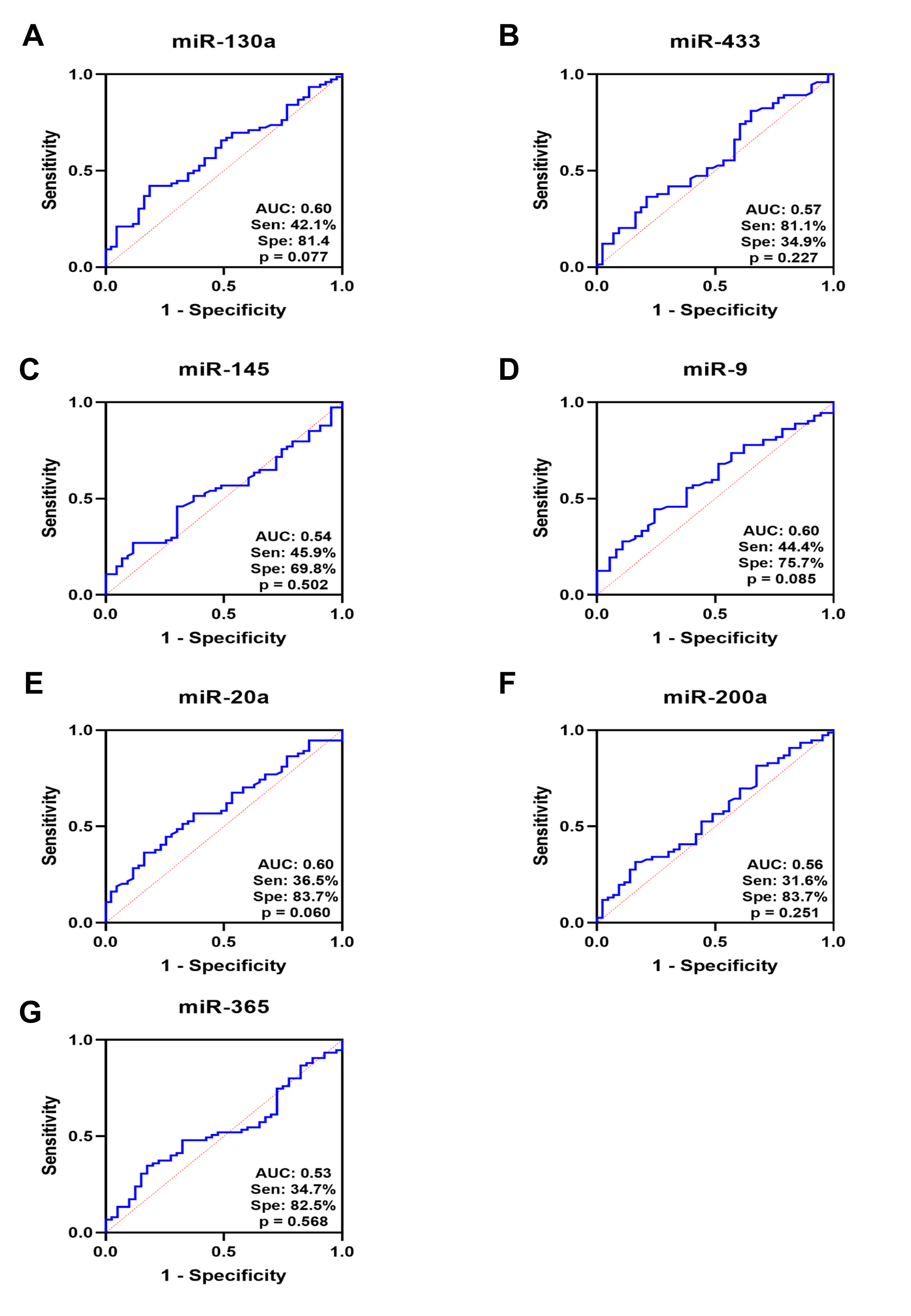

Supplement: Supplementary Figure 8 — Receiver Operating Characteristics (ROC) curves for (A) miR-130a, (B) miR-433, (C) miR-145, (D) miR-9, (E) miR-20a, (F) miR-200a, and (G) miR-365. * p < 0.05. [file Image8.tif]

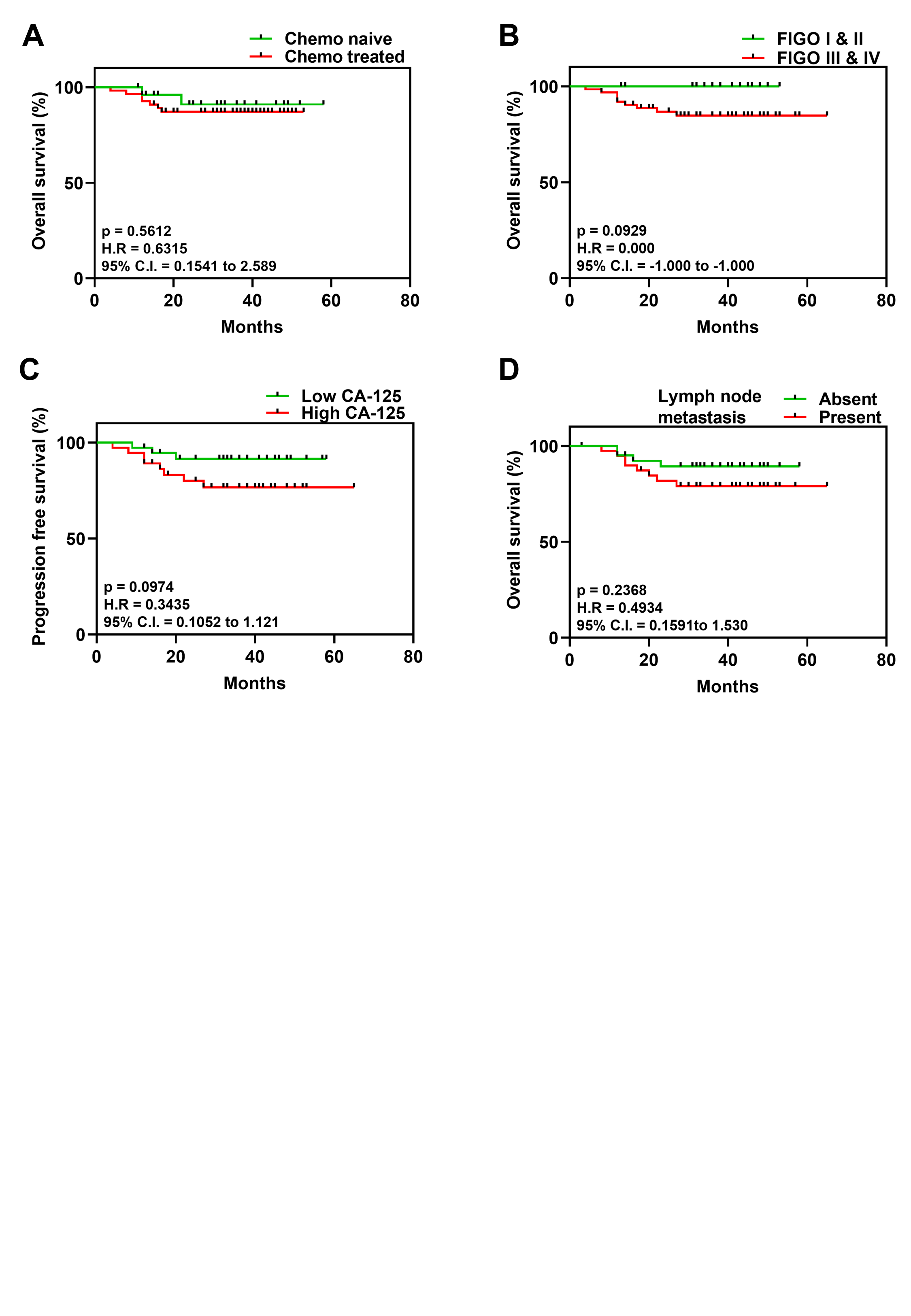

Supplement: Supplementary Figure 9 — Kaplan Meier curves displaying the association of (A) mode of treatment, (B) FIGO Stage of the disease, (C) serum levels of CA-125 and (D) presence or absence of lymph node metastasis in ovarian cancer patients and their respective overall survival. * p < 0.05. [file Image9.tif]
