# Supplementary material for: Chemotherapy-induced alterations in miRNA expression and their prognostic implications in ovarian cancer
Source: Front Oncol. 2025 Aug 22;15:1580565. doi: 10.3389/fonc.2025.1580565 (PMC12411204; doi:10.3389/fonc.2025.1580565)
Supplement: Supplementary file 10 [file Table1.docx]

|  |  |
| --- | --- |

**Table S1-**

The statistical distribution of miRNA expression in serum and tissue of the populations under study

(a)

| Serum expression | Healthy control | | | Chemonaive OvCA | | | Chemotreated OvCA | | |
| --- | --- | --- | --- | --- | --- | --- | --- | --- | --- |
|  | **Mean** | **SD** | **SEM** | **Mean** | **SD** | **SEM** | **Mean** | **SD** | **SEM** |
| miR-182 | -0.34 | 3.96 | 0.58 | -4.37 | 4.19 | 0.87 | -1.72 | 3.24 | 0.49 |
| miR-433 | -2.32 | 3.40 | 0.52 | -0.46 | 4.15 | 0.87 | -1.26 | 3.48 | 0.49 |
| miR-145 | -4.36 | 3.15 | 0.46 | -4.82 | 3.94 | 0.82 | -2.50 | 3.44 | 0.50 |
| miR-20a | -6.77 | 3.64 | 0.56 | -6.51 | 4.88 | 1.02 | -4.15 | 3.70 | 0.53 |
| miR-23b | -9.07 | 4.71 | 0.73 | -11.06 | 3.29 | 0.69 | -11.08 | 4.67 | 0.65 |
| miR-106a | -6.29 | 4.16 | 0.68 | -9.87 | 5.14 | 1.15 | -10.12 | 5.77 | 0.84 |
| miR-130a | 3.47 | 3.03 | 0.46 | 1.26 | 4.06 | 0.85 | 2.31 | 4.45 | 0.61 |
| miR-200a | 1.31 | 2.79 | 0.44 | -1.27 | 3.97 | 0.83 | 0.47 | 3.53 | 0.49 |
| miR-9 | -7.24 | 3.21 | 0.52 | -5.79 | 3.60 | 0.77 | -7.02 | 4.26 | 0.61 |
| miR-365 | -5.41 | 3.52 | 0.56 | -4.06 | 5.25 | 1.10 | -5.35 | 4.57 | 0.63 |

(b)

| Tissue expression | Chemonaive OvCA | | | Chemotreated OvCA | | |
| --- | --- | --- | --- | --- | --- | --- |
|  | **Mean** | **SD** | **SEM** | **Mean** | **SD** | **SEM** |
| miR-182 | -2.32 | 3.40 | 0.80 | -4.03 | 2.45 | 0.38 |
| miR-433 | -9.92 | 3.41 | 0.83 | -9.07 | 5.79 | 0.85 |
| miR-145 | -4.67 | 3.87 | 0.94 | -4.46 | 6.14 | 0.92 |
| miR-20a | -5.67 | 6.05 | 1.43 | -5.94 | 5.29 | 0.85 |
| miR-23b | -3.67 | 2.88 | 0.72 | -6.36 | 4.86 | 0.79 |
| miR-106a | -6.62 | 4.30 | 1.04 | -6.70 | 4.58 | 0.71 |
| miR-130a | -10.60 | 3.91 | 0.95 | -9.31 | 6.04 | 0.92 |
| miR-200a | 2.05 | 5.85 | 1.42 | 2.97 | 7.33 | 1.11 |
| miR-9 | -6.43 | 5.98 | 1.49 | -10.08 | 6.76 | 1.07 |
| miR-365 | 1.30 | 7.25 | 1.71 | 3.05 | 8.69 | 1.26 |

**Table S2**

Survival analysis of ovarian cancer patients based on the miRNA expression levels in the serum

| **PFS** | | | | | **OS** | | | |
| --- | --- | --- | --- | --- | --- | --- | --- | --- |
|  | **p-value** | **Hazard ratio** | **95% C.I.** | **χ^2^** | **p-value** | **Hazard ratio** | **95% C.I.** | **χ^2^** |
| **miR-182** | 0.4299 | 1.28 | 0.68 to 2.40 | 0.623 | 0.2905 | 2.34 | 0.53 to 10.33 | 1.117 |
| **miR-130a** | 0.059 | 1.84 | 0.98 to 3.45 | 3.567 | 0.2771 | 2.4 | 0.54 to 10.56 | 1.181 |
| **miR-106a** | 0.9471 | 0.97 | 0.48 to 1.95 | 0.004 | 0.4719 | 1.83 | 0.37 to 9.11 | 0.5175 |
| **miR-200a** | 0.1792 | 1.52 | 0.81 to 2.85 | 1.804 | 0.7581 | 1.26 | 0.28 to 5.55 | 0.094 |
| **miR-9** | 0.116 | 1.64 | 0.87 to 3.12 | 2.47 | 0.2867 | 2.36 | 0.53 to 10.39 | 1.135 |
| **miR-23b** | 0.6079 | 0.85 | 0.45 to 1.59 | 0.263 | 0.6454 | 0.70 | 0.16 to 3.11 | 0.211 |
| **miR-433** | 0.533 | 1.21 | 0.64 to 2.27 | 0.388 | 0.6317 | 0.69 | 0.15 to 3.06 | 0.229 |
| **miR-145** | 0.1847 | 1.51 | 0.80 to 2.84 | 1.76 | 0.6864 | 1.35 | 0.30 to 5.97 | 0.163 |
| **miR-365** | 0.7391 | 0.89 | 0.47 to 1.69 | 0.110 | 0.2343 | 2.59 | 0.58 to 11.40 | 1.415 |
| **miR-20a** | 0.5233 | 1.22 | 0.65 to 2.29 | 0.407 | 0.7967 | 1.21 | 0.27 to 5.35 | 0.066 |

**Table S3-**

Survival analysis of ovarian cancer patients based on the miRNA expression levels in the tumor tissues

|  | **PFS** | | | |  | **OS** | | | |
| --- | --- | --- | --- | --- | --- | --- | --- | --- | --- |
|  | **p value** | **Hazard ratio** | **95% C.I.** | **χ^2^** |  | **p value** | **Hazard ratio** | **95% C.I.** | **χ^2^** |
| **miR-182** | 0.9417 | 0.974 | 0.48 to 1.97 | 0.005 |  | 0.4665 | 1.856 | 0.37 to 9.20 | 0.530 |
| **miR-130a** | 0.1753 | 0.633 | 0.30 to 1.31 | 1.837 |  | 0.4658 | 1.858 | 0.37 to 9.21 | 0.532 |
| **miR-106a** | 0.3684 | 0.728 | 0.34 to 1.53 | 0.809 |  | 0.6083 | 1.55 | 0.30 to 7.78 | 0.262 |
| **miR-200a** | 0.3792 | 0.731 | 0.34 to 1.55 | 0.773 |  | 0.8657 | 0.871 | 0.17 to 4.33 | 0.028 |
| **miR-9** | 0.3856 | 0.721 | 0.34 to 1.49 | 0.752 |  | 0.3037 | 2.362 | 0.47 to 11.77 | 1.058 |
| **miR-23b** | 0.1976 | 0.640 | 0.32 to 1.24 | 1.66 |  | 0.4292 | 1.807 | 0.40 to 8.09 | 0.624 |
| **miR-433** | 0.587 | 1.206 | 0.60 to 2.39 | 0.295 |  | 0.1104 | 4.832 | 0.97 to 23.95 | 2.549 |
| **miR-145** | 0.8938 | 1.046 | 0.52 to 2.08 | 0.017 |  | 0.4391 | 1.926 | 0.38 to 9.54 | 0.598 |
| **miR-365** | 0.9233 | 0.968 | 0.48 to 1.92 | 0.009 |  | 0.9105 | 0.912 | 0.18 to 4.52 | 0.0126 |
| **miR-20a** | 0.5111 | 0.795 | 0.38 to 1.64 | 0.431 |  | 0.7036 | 1.411 | 0.24 to 8.15 | 0.144 |

**Table S4-**

Correlation analysis of serum miRNA expression levels with the clinical parameters of ovarian cancer patients

| **Parameters** | | **Serum** | | | | | | | | | |
| --- | --- | --- | --- | --- | --- | --- | --- | --- | --- | --- | --- |
|  |  | **miR-182** | **miR-433** | **miR-145** | **miR-9** | **miR-20a** | **miR-130a** | **miR-23b** | **miR-200a** | **miR-365** | **miR-106a** |
| **Age** | **r** | -0.011 | 0.013 | 0.066 | 0.024 | 0.051 | 0.110 | 0.038 | 0.120 | 0.110 | 0.037 |
|  | **95% C.I.** | -0.25 to 0.23 | -0.23 to 0.26 | -0.18 to 0.30 | -0.22 to 0.27 | -0.19 to 0.29 | -0.14 to 0.34 | -0.21 to 0.28 | -0.12 to 0.35 | -0.13 to 0.35 | -0.22 to 0.29 |
|  | **p value** | 0.928 | 0.917 | 0.588 | 0.850 | 0.678 | 0.381 | 0.752 | 0.310 | 0.346 | 0.776 |
| **Stage** | **r** | -0.14 | -0.13 | -0.12 | -0.043 | 0.062 | -0.23 | 0.036 | -0.18 | -0.18 | 0.15 |
|  | **95% C.I.** | -0.38 to 0.11 | -0.37 to 0.12 | -0.36 to 0.13 | -0.29 to 0.21 | -0.19 to 0.31 | -0.45 to 0.016 | -0.21 to 0.28 | -0.41 to 0.074 | -0.41 to 0.068 | -0.12 to 0.40 |
|  | **p value** | 0.251 | 0.300 | 0.328 | 0.739 | 0.621 | 0.059 | 0.772 | 0.153 | 0.139 | 0.258 |
| **CA-125** | **r** | 0.028 | -0.076 | 0.071 | -0.087 | 0.038 | 0.011 | -0.12 | -0.039 | -0.048 | 0.31 |
|  | **95% C.I.** | -0.24 to 0.29 | -0.33 to 0.19 | -0.20 to 0.33 | -0.35 to 0.19 | -0.23 to 0.30 | -0.25 to 0.27 | -0.37 to 0.15 | -0.30 to 0.22 | -0.31 to 0.22 | 0.037 to 0.55 |
|  | **p value** | 0.831 | 0.570 | 0.592 | 0.520 | 0.777 | 0.935 | 0.368 | 0.763 | 0.717 | **0.023** |
| **Lymph node metastasis** | **r** | -0.14 | -0.054 | -0.013 | 0.029 | 0.1 | -0.21 | -0.23 | -0.093 | -0.097 | -0.006 |
|  | **95% C.I.** | -0.39 to 0.12 | -0.31 to 0.21 | -0.27 to 0.25 | -0.24 to 0.29 | -0.17 to 0.35 | -0.45 to 0.046 | -0.46 to 0.032 | -0.34 to 0.17 | -0.35 to 0.17 | -0.28 to 0.27 |
|  | **p value** | 0.277 | 0.682 | 0.924 | 0.830 | 0.446 | 0.095 | 0.076 | 0.471 | 0.459 | 0.965 |
| **Ascites** | **r** | 0.19 | -0.023 | 0.18 | -0.031 | 0.075 | 0.13 | 0.2 | 0.21 | 0.12 | 0.13 |
|  | **95% C.I.** | -0.07 to 0.42 | -0.28 to 0.24 | -0.08 to 0.41 | -0.29 to 0.23 | -0.19 to 0.33 | -0.13 to 0.37 | -0.06 to 0.43 | -0.04 to 0.44 | -0.14 to 0.36 | -0.14 to 0.39 |
|  | **p value** | 0.147 | 0.860 | 0.170 | 0.812 | 0.563 | 0.309 | 0.122 | 0.089 | 0.369 | 0.328 |

**Table S5-** Correlation analysis of tissue miRNA expression levels with the clinical parameters of ovarian cancer patients

| **Parameters** | | **Tissue** | | | | | | | | | |
| --- | --- | --- | --- | --- | --- | --- | --- | --- | --- | --- | --- |
|  |  | **miR-182** | **miR-433** | **miR-145** | **miR-9** | **miR-20a** | **miR-130a** | **miR-23b** | **miR-200a** | **miR-365** | **miR-106a** |
| **Age** | **r** | 0.140 | 0.180 | 0.053 | -0.008 | 0.100 | 0.160 | 0.310 | 0.130 | 0.055 | 0.120 |
|  | **95% C.I.** | -0.14 to 0.39 | -0.089 to 0.42 | -0.22 to 0.31 | -0.28 to 0.26 | -0.17 to 0.37 | -0.11 to 0.41 | 0.032 to 0.54 | -0.15 to 0.38 | -0.21 to 0.31 | -0.15 to 0.38 |
|  | **p value** | 0.319 | 0.176 | 0.691 | 0.953 | 0.446 | 0.237 | **0.026** | 0.350 | 0.674 | 0.365 |
| **Stage** | **r** | 0.17 | 0.061 | 0.043 | -0.11 | 0.16 | 0.13 | 0.006 | 0.064 | 0.013 | 0.21 |
|  | **95% C.I.** | -0.12 to 0.44 | -0.22 to 0.34 | -0.24 to 0.32 | -0.39 to 0.19 | -0.14 to 0.43 | -0.16 to 0.41 | -0.30 to 0.31 | -0.23 to 0.34 | -0.26 to 0.28 | -0.089 to 0.47 |
|  | **p value** | 0.237 | 0.669 | 0.765 | 0.458 | 0.272 | 0.355 | 0.969 | 0.659 | 0.926 | 0.156 |
| **CA-125** | **r** | 0.3 | 0.31 | 0.18 | -0.14 | 0.33 | 0.35 | 0.15 | 0.25 | 0.29 | 0.25 |
|  | **95% C.I.** | 0.019 to 0.53 | 0.037 to 0.53 | -0.10 to 0.43 | -0.41 to 0.14 | 0.051 to 0.56 | 0.081 to 0.58 | -0.15 to 0.42 | -0.033 to 0.49 | 0.028 to 0.52 | -0.033 to 0.50 |
|  | **p value** | **0.032** | **0.023** | 0.193 | 0.315 | **0.018** | **0.010** | 0.306 | 0.075 | **0.026** | 0.074 |
| **Lymph node metastasis** | **r** | 0.22 | 0.21 | 0.23 | 0.0016 | 0.25 | 0.24 | 0.22 | 0.21 | 0.088 | 0.09 |
|  | **95% C.I.** | -0.082 to 0.48 | -0.083 to 0.47 | -0.063 to 0.48 | -0.29 to 0.30 | -0.053 to 0.52 | -0.056 to 0.50 | -0.10 to 0.50 | -0.091 to 0.48 | -0.19 to 0.36 | -0.22 to 0.38 |
|  | **p value** | 0.142 | 0.148 | 0.112 | 0.992 | 0.094 | 0.100 | 0.165 | 0.158 | 0.525 | 0.556 |
| **Ascites** | **r** | 0.086 | -0.066 | -0.055 | -0.0053 | 0.011 | 0.11 | 0.038 | 0.062 | 0.021 | 0.022 |
|  | **95% C.I.** | -0.20 to 0.36 | -0.33 to 0.21 | -0.33 to 0.22 | -0.29 to 0.28 | -0.27 to 0.29 | -0.17 to 0.38 | -0.26 to 0.33 | -0.22 to 0.33 | -0.25 to 0.29 | -0.26 to 0.30 |
|  | **p value** | 0.542 | 0.632 | 0.694 | 0.970 | 0.939 | 0.425 | 0.799 | 0.657 | 0.874 | 0.877 |

**Table S6**

Correlation analysis between the serum and tissue miRNA expression levels of ovarian cancer patients

| miRNA | r | 95% C.I. | p value |
| --- | --- | --- | --- |
| miR-182 | 0.12 | -0.16 to 0.39 | 0.38 |
| miR-130a | 0.20 | -0.078 to 0.46 | 0.14 |
| miR-200a | 0.28 | 0.0078 to 0.52 | **0.04** |
| miR-106a | 0.14 | -0.17 to 0.43 | 0.35 |
| miR-9 | -0.22 | -0.47 to 0.066 | 0.12 |
| miR-433 | 0.06 | -0.22 to 0.33 | 0.67 |
| miR-145 | 0.12 | -0.16 to 0.38 | 0.38 |
| miR-20a | 0.06 | -0.22 to 0.34 | 0.65 |
| miR-23b | -0.01 | -0.30 to 0.28 | 0.95 |
| miR-365 | -0.17 | -0.42 to 0.096 | 0.19 |
